# Neuronal double-stranded DNA accumulation induced by DNase II deficiency drives tau phosphorylation and neurodegeneration

**DOI:** 10.1186/s40035-024-00427-8

**Published:** 2024-08-02

**Authors:** Ling-Jie Li, Xiao-Ying Sun, Ya-Ru Huang, Shuai Lu, Yu-Ming Xu, Jing Yang, Xi-Xiu Xie, Jie Zhu, Xiao-Yun Niu, Dan Wang, Shi-Yu Liang, Xiao-Yu Du, Sheng-Jie Hou, Xiao-Lin Yu, Rui-Tian Liu

**Affiliations:** 1 State Key Laboratory of Biochemical Engineering, Institute of Process Engineering, Chinese Academy of Sciences, Beijing, 100190 China; 2 University of Chinese Academy of Sciences, Beijing, 100049 China; 3 Department of Neurology, the First Affiliated Hospital of Zhengzhou University, Zhengzhou, 450052 China; 4 College of Life Science, Ningxia University, Yinchuan, 750021 China; 5 Department of BigData, Beijing Medintell Bioinformatic Technology Co., LTD, Beijing, 100081 China

**Keywords:** DNase II, Alzheimer’s disease, Double-stranded DNA, Tau phosphorylation, Tauopathy

## Abstract

**Background:**

Deoxyribonuclease 2 (DNase II) plays a key role in clearing cytoplasmic double-stranded DNA (dsDNA). Deficiency of DNase II leads to DNA accumulation in the cytoplasm. Persistent dsDNA in neurons is an early pathological hallmark of senescence and neurodegenerative diseases including Alzheimer’s disease (AD). However, it is not clear how DNase II and neuronal cytoplasmic dsDNA influence neuropathogenesis. Tau hyperphosphorylation is a key factor for the pathogenesis of AD. The effect of DNase II and neuronal cytoplasmic dsDNA on neuronal tau hyperphosphorylation remains unclarified.

**Methods:**

The levels of neuronal DNase II and dsDNA in WT and Tau-P301S mice of different ages were measured by immunohistochemistry and immunolabeling, and the levels of DNase II in the plasma of AD patients were measured by ELISA. To investigate the impact of DNase II on tauopathy, the levels of phosphorylated tau, phosphokinase, phosphatase, synaptic proteins, gliosis and proinflammatory cytokines in the brains of neuronal DNase II-deficient WT mice, neuronal DNase II-deficient Tau-P301S mice and neuronal DNase II-overexpressing Tau-P301S mice were evaluated by immunolabeling, immunoblotting or ELISA. Cognitive performance was determined using the Morris water maze test, Y-maze test, novel object recognition test and open field test.

**Results:**

The levels of DNase II were significantly decreased in the brains and the plasma of AD patients. DNase II also decreased age-dependently in the neurons of WT and Tau-P301S mice, along with increased dsDNA accumulation in the cytoplasm. The DNA accumulation induced by neuronal DNase II deficiency drove tau phosphorylation by upregulating cyclin-dependent-like kinase-5 (CDK5) and calcium/calmodulin activated protein kinase II (CaMKII) and downregulating phosphatase protein phosphatase 2A (PP2A). Moreover, DNase II knockdown induced and significantly exacerbated neuron loss, neuroinflammation and cognitive deficits in WT and Tau-P301S mice, respectively, while overexpression of neuronal DNase II exhibited therapeutic benefits.

**Conclusions:**

DNase II deficiency and cytoplasmic dsDNA accumulation can initiate tau phosphorylation, suggesting DNase II as a potential therapeutic target for tau-associated disorders.

**Graphical Abstract:**

Scheme depicting the possible mechanism by which DNase II deficiency induces cognitive impairment in mice. DNase II deficiency induces tau phosphorylation by regulating kinases CDK5 and CaMKII as well as phosphatase PP2A through accumulation of undigested damaged DNA in the cytoplasm of neurons. Then phosphorylated tau induces synaptic loss, neuroinflammation, and neuronal apoptosis, eventually rendering cognitive impairment in mice.

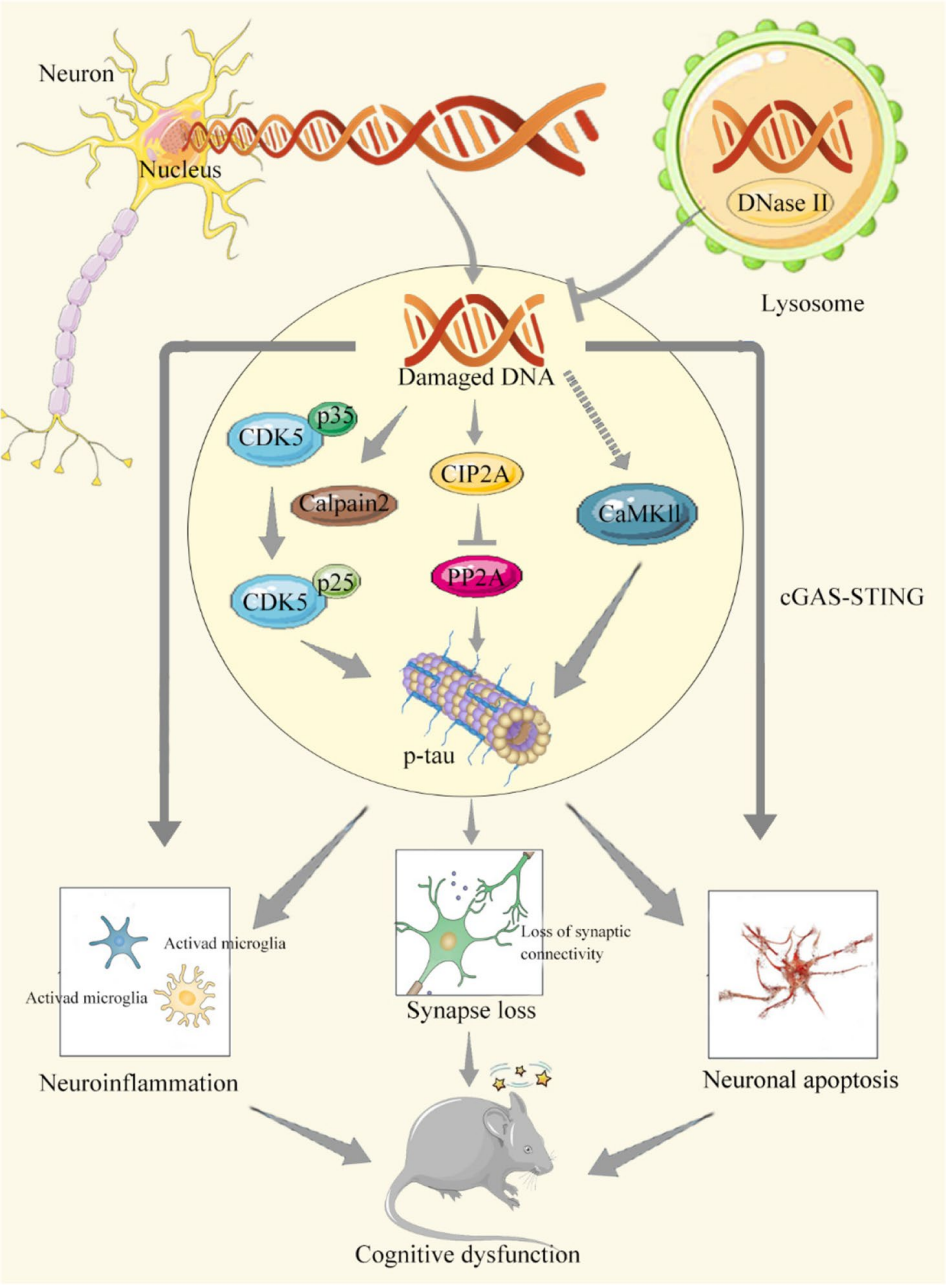

**Supplementary Information:**

The online version contains supplementary material available at 10.1186/s40035-024-00427-8.

## Background

Deoxyribonuclease 2 (DNase II) is one of the deoxyribonucleases localized in lysosomes and targets double-stranded DNA (dsDNA), including nuclear and mitochondrial DNA, for degradation [1–4]. In healthy cells, damaged and irreparable nuclear DNA fragments are trafficked to the cytosol, delivered to lysosomes, and hydrolyzed by DNase II before they accumulate [5]. DNase II deficiency in leukocytes leads to the accumulation of undigested DNA in the cytoplasm and causes autoinflammation mediated by type-I-interferon (IFN-I) [6]. In senescent cells, DNase II downregulation provokes the senescence-associated secretory phenotype through aberrant activation of cytoplasmic DNA sensors and the cyclic GMP-AMP synthase (cGAS)–stimulator of interferon genes (STING) pathway with cytoplasmic accumulation of DNA [7–10]. Although the role of DNase II in peripheral systems has been extensively studied, its role in the central nervous system is relatively poorly understood.

In the central nervous system, neurons are under great potential threats of DNA damage due to oxidative and metabolic stress from the high demand of oxygen, which requires efficient DNA damage surveillance, repair and clearance [11, 12]. Increasing evidence indicates that the accumulation of damaged DNA increases with age [13, 14], and dsDNA breaks, the most severe form of DNA damage induced by ROS, ionizing radiation, genotoxic compounds as well as physiological brain activity, are also increased in neurons [15–17] and in AD patients and AD models [18, 19]. dsDNA breaks are demonstrated to occur commonly in the brain [16, 18, 20], which exacerbates cellular senescence and various neurological disorders. However, the role of DNase II and cytoplasmic damaged DNA in AD pathology remains unclarified.

Tau is a microtubule-binding protein that maintains polymerization and stabilization of microtubules in neuronal axons and dendrites to ensure essential cargo transportation. However, under pathological states, tau hyperphosphorylation increases, detaches from microtubules and aggregates into oligomers and paired helical filaments, causing neuronal dysfunction such as autophagy dysfunction, mitochondrial dysfunction, and synaptic impairment, and finally resulting in neuronal degeneration [21]. Hyperphosphorylated tau protein is a pathological hallmark of several neurodegenerative diseases such as AD [22], frontotemporal dementia [23] and progressive supranuclear palsy [24]. Tau phosphorylation is regulated by multiple kinases and phosphatases, such as glycogen synthase kinase-3β (GSK-3β), CDK5, cell cycle checkpoint kinases (Chk1, Chk2), CaMKII, and protein phosphatases PP2A [21, 22, 25]. However, regulation of the activity of these enzymes remains far from complete.

In this study, we set out to examine the levels of neuronal DNase II and cytoplasmic damaged DNA in the brains of WT mice, Tau-P301S mice and AD patients, explore the roles of neuronal DNase II and cytoplasmic damaged DNA in tau phosphorylation, neuronal apoptosis and mouse cognition, and investigate the therapeutic effect in vivo by increasing DNase II expression.

## Materials and methods

### Subjects and sample collection

This study was approved by the Institutional Review Board of the First Affiliated Hospital of Zhengzhou University Ethics Committee. All participants signed written informed consent before their enrollment in the study. We collected plasma samples from patients with mild cognitive impairment (MCI) (*n* = 14) with a median age of 73 years (range, 57–84 years), AD patients (*n* = 15) with a median age of 72 years (range, 57–85 years), and 13 control subjects with a median age of 71 years (range, 65–78 years). Patient’s information including age, sex and clinical diagnosis is shown in Additional file 1: Table S1. All diagnoses of AD in this study were based on the recommendations of the National Institute on Aging and the Alzheimer’s Association workgroup [26] or National Institute of Neurological and Communicative Disorders and Stroke and the Alzheimer’s Disease and Related Disorders Association criteria [27]. The level of DNase II in human plasma was determined by Human DNASE2A (Deoxyribonuclease-2-alpha) ELISA Kit (HZBIO, Shanghai, China, #HZE52913h) according to the manufacturer’s instructions.

### Animal treatment

Three-month-old male C57BL/6 mice were purchased from Si Pei Fu (Beijing) Biotechnology Co. Ltd. (Beijing, China). Tau-P301S mice were originally obtained from Jackson Laboratory (The Jackson Laboratory, Bar harbor, ME, Stock #008169). Three- and 9-month-old male Tau-P301S mice were generated by breeding. All mice were group-housed with free access to food and water, and kept in a colony room at 22 ± 2℃ and 45% ± 10% humidity on a reverse 12 h light/dark cycle. All animal experiments were performed in accordance with the China Public Health Service Guide for the Care and Use of Laboratory Animals. Experiments involving mice and protocols were approved by the Institutional Animal Care and Use Committee of Tsinghua University. The 3-month-old WT or Tau-P301S mice were divided into two groups (*n* = 10 mice per group) to receive injection of AAV-shDNase2a (WT-KD or Tau-KD) or control virus (WT-CON or Tau-CON), respectively. The 9-month-old Tau-P301S mice were divided into two groups (*n* = 8 mice per group) to receive injection of AAV-overexpression DNase2a (Tau-OVER) or control virus (Tau-CON).

### Primary neuronal culture

Primary hippocampal neurons were obtained from the hippocampi of C57BL/6 mouse embryos on embryonic days 16–17 (E16–17). Briefly, hippocampi were dissected and digested with 0.25% trypsin containing 1 mg/ml DNase I (Thermo Fisher Scientific, Waltham, MA, #90083), and then the dissociated cells were plated on poly-*D*-lysine-coated coverslips at a density of 100,000–300,000 cells/well in a 12-well dish and cultured in neurobasal medium supplemented with B27 (Gibco, Waltham, MA, #17504–044), GlutaMAX (Gibco, #35050,061), 0.5% penicillin–streptomycin (Gibco, #15070063) and Cytarabine (MedChemExpress, Monmouth Junction, NJ, #HY-13605) for 7–10 days in vitro (DIV 7–10). The medium was half-exchanged with fresh medium three times a week. Neuronal maturation was determined by immunocytochemistry with anti-MAP2 and anti-NeuN antibodies.

### Cell culture

293 T cells were purchased from ATCC (Rockville, MD, CRL-3216) and cultured in DMEM medium (Gibco, #C11965500CP) supplemented with 10% fetal bovine serum (Gibco, #10099141) and 0.5% penicillin–streptomycin in 5% CO_2_ at 37 °C.

### Analysis of mRNA levels of DNase II in AD patients

The Gene Expression Omnibus (GEO) Series (GSE) matrix files were downloaded from GEO of the National Center for Biotechnology Information (NCBI). In the dataset, there were 8 normal controls, 20 early-onset AD cases (EOAD, age-at-onset less than 60 years) and 19 late-onset AD cases (LOAD, age-at-onset between 70 and 80 years). All samples were collected from different research centers and preserved at −80 °C until RNA preparation. The dataset was analyzed using DESeq2 in R package (3.6.3) and Mann–Whitney test was used to analyze the expression of DNase II in AD patients and the control group.

### Lentivirus production and infection

All shRNAs were chemically synthesized by Invitrogen (Frederick, MD) and inserted into the pSicoR from Addgene (Watertown, MA). Lentiviral package plasmids (Addgene) and the pSicoR were transfected into 293 T cells by Zlip2000 (Beijing Zoman Biotechnology, Beijing, China, #ZC302-2) according to the instructions. The supernatant containing lentivirus was collected at 48 h after transfection and centrifuged at 3000 rpm for 5 min to remove the cell debris and further ultracentrifuged to obtain high titer stocks. The pellet was resuspended in phosphate buffer saline (PBS) and stored at −80 °C. The final viral concentration was calculated by qPCR assay. The shRNA sequence is as follows: shDNase2a, GGGTCTAGGGATACTCCAAAG.

To explore the effect of DNase II on the expression of phosphorylated tau and the cGAS–STING pathway-related proteins, the expression of DNase II was reduced in primary hippocampal neurons (DIV 7–10) by infection with shDNase2a. Neurons were analyzed 72 h after infection (DIV 10–13). Meanwhile, at 48 h after infection, the cells were treated with 2.5 μM roscovitine [28] (MedChemExpress, #HY-30237) for 24 h to inhibit CDK5, or 5 μM DT061 [29] (MedChemExpress, #HY-112929) for 24 h to activate PP2A. Then the cells were harvested in an ice-cold RIPA lysis solution (MedChemExpress, #HY-K1001) with 1 mM phenylmethylsulfonyl fluoride, protease inhibitor cocktail set I (Millipore, Billerica, MA, #539131), and 1% (*v*/*v*) phosphatase inhibitor cocktails (Solarbio Left Sciences, Beijing, China, #P1260). The cell lysates were centrifuged for 10 min at 13,000 × *g*, and the supernatants were isolated and stored for analysis. For immunofluorescence assay, cells were harvested and fixed in 4% paraformaldehyde (PFA).

### Stereotaxic injection of AAV

Mice were deeply anesthetized by intraperitoneal injection of a mixture of ketamine (100 mg/kg) and xylazine (10 mg/kg). To knockdown neuronal DNase II, 4 μl (8 × 10^12^ vg/ml) of AAV-shDNase2a or AAV-shCON was injected bilaterally in 3-month-old WT and TauP301S mice at the following target coordinates: anteroposterior (AP) − 2.06 mm, mediolateral (ML) ± 1.5 mm, and dorsoventral (DV) − 1.5 mm (CA1)/ − 2 mm (DG) from bregma (Fig. S4b). To overexpress neuronal DNase II, 9-month-old TauP301S mice received bilateral injection of 4 μl (6 × 10^12^ vg/mL) of AAV-DNase2a or AAV-CON at the same target sites. The rate of injection was 0.5 μl/min and the needle remained in place for an additional 5 min after injection. After surgery, the surgical sites were cleaned with sterile saline and the incision was sutured. All mice were monitored and post-surgical care was provided. Four weeks later, the mice underwent behavioral tests, and then sacrificed for biochemical and histological analysis.

The sequences of shDNase2a and shCON were 5’-GGGTCTAGGGATACTCCAAAG-3’, and 5’-GAAGTCGTGAGAAGTAGAA-3’, respectively. AAV was purchased from Obio Technology (Shanghai, China) with a serotype of AAV2/9.

### Morris water maze (MWM) test

The MWM test was performed as described previously with minor modifications [30]. Briefly, the water maze consisted of a pool (120 cm in diameter) with opaque water (22 ± 1 °C) and a platform (10 cm in diameter) submerged 1.0 cm under the water. In the training phase (days 1–5), mice were allowed to swim for 60 s to find the platform, and stay there for 20 s. Mice unable to locate the platform were manually guided to it. All mice were trained twice a day over five consecutive days, with an interval of 3–4 h. Twenty hours after the last training trial, the mice were tested for memory retention in a probe trial in the absence of the platform. The duration of the probe trial was 60 s. The swimming activity of each mouse was recorded by the video camera (Sony, Tokyo, Japan).

### Forced Y-maze test

The Y-maze was made up of three identical arms (8 cm × 30 cm × 15 cm) covered with black paper and separated by an angle of 120°. The test consisted of 2 trials with an interval of 1 h. For the training trial, each mouse was allowed to explore freely only 2 arms (the start and the familiar arms) of the maze for 10 min, and the third arm (the new arm) was blocked. For the testing trial, the mouse was put back in the same starting arm as in the training trial with free access to all three arms for 5 min. All trials were recorded by a video system, and the number of entries and the time spent in each arm were analyzed. The arms were cleaned with 75% ethanol solution between trials.

### Spontaneous Y-maze test

The spontaneous Y-maze test aims to assess the short-term memory performance. The maze was the same as the forced Y-maze, except that the marker at the end of each arm was changed to eliminate the effects of the former forced Y-maze. This test consisted of a single 5-min trial in which the mouse was allowed to move freely to all three arms of the Y-maze. The series of arm entries, including possible returns into the same arm, were recorded with a camera connected to a computer. An alternation was defined as entry into all three arms on consecutive occasions. The number of maximum alternations was therefore the total number of arm entries minus 2, and the percentage of alternations was calculated as (actual alternations/maximum alternations) × 100%.

### Novel object recognition (NOR) test

Briefly, in the habituation phase, each mouse was allowed to freely explore the behavioral arena (50 cm × 50 cm × 25 cm white plastic box, empty) for 5 min the day before testing. For the training session, a mouse was placed in the box having two identical objects in the upper two corners and allowed to explore for 5 min. After a 6-h interval, the mouse was allowed to explore one familiar and one novel object for 5 min in the same box in the test session. The time spent exploring and sniffing each object was recorded. The results were expressed as the discrimination index, which refers to: (Time_novel_ − Time_old_) / (Time_novel_ + Time_old_). The box was cleaned with 75% alcohol between trials to eliminate olfactory cues.

### Open field test

Mice were placed individually in the center of the chamber (27 × 27 × 20.3 cm^3^) equipped with a camera (Sony, Tokyo, Japan). Their free behavior was recorded for 10 min. Velocity, center duration and total distance were quantitatively analyzed. Chamber was cleaned with 75% ethanol between trials.

### Immunocytochemistry

Cells were washed with PBS three times, fixed in 4% PFA for 20 min at room temperature, permeabilized with 0.3% Triton X-100 for 30 min and blocked with 10% donkey serum albumin (DSA) in PBS for 1 h at room temperature. Then the cells were incubated with primary antibodies overnight at 4 ℃ followed by corresponding fluorescently-conjugated (-488, -594 or -647) secondary antibodies for 45 min, counterstained with DAPI (1:10,000) for 15 min at room temperature in dark, and then mounted on coverslips with anti-fade mounting medium. Fluorescence signals were captured on a laser scanning confocal microscope (Leica TCS SP8, Germany). Moreover, a fluorescent terminal deoxynucleotidyl transferase nick-end labeling kit was used for terminal deoxynucleotidyl transferase mediated dUTP nick-end labeling (TUNEL) staining (Roche, Basel, Switzerlan, #12156792910).

### Immunohistochemistry

Mice were deeply anesthetized by intraperitoneal injection of a mixture of ketamine (100 mg/kg) and xylazine (10 mg/kg) and perfused with ice-cold PBS containing heparin (10 U/ml) before sacrifice. Mouse brains were immediately removed and divided along the sagittal plane. The left brain hemisphere was fixed in 4% PFA at 4 ℃ overnight and processed for paraffin-embedded sections. For immunohistochemistry analysis, 5-μm coronal paraffin-embedded serial sections were deparaffinized and subjected to antigen retrieval using citrate buffer (0.01 M, pH 6.0, 0.05% Tween-20) at 95 °C for 20 min. The sections were then incubated with 0.3% H_2_O_2_ and washed 3 times with 1 × PBS. Sections were then permeabilized and blocked with 10% goat serum albumin or DSA in 0.3% Triton-X 100 for 1 h at room temperature. Then sections were incubated with the primary antibodies overnight at 4 °C, followed by corresponding secondary antibodies conjugated to Alexa Fluor 488, 594 or 647. The sections were imaged on the Leica TCS SP8 confocal microscope. For 3’-diaminobenzidine (DAB) immunostaining, the sections were incubated with a corresponding HRP-labeled secondary antibody and visualized with DAB by an Olympus IX73 inverted microscope with DP80 camera. All images were analyzed by Image J software (v1.52a). Moreover, a fluorescent terminal deoxynucleotidyl transferase nick-end labeling kit (Roche, Basel, Switzerlan, #12156792910) was used for TUNEL staining.

The following primary antibodies were used for immunohistochemistry and immunocytochemistry: anti-MAP2 antibody (Invitrogen, #PA1-16751, 1:500), anti-NeuN antibody (Abcam, Cambridge, UK, #ab177487, 1:200), anti-ionized calcium-binding adapter molecule 1 (IBA1) antibody (Genetex, San Antonio, TX, #GTX101495, 1:200), anti-GFAP antibody (Cell Signaling Technology, Boston, MA, #3670S, 1:200), anti-DNase II antibody (Proteintech, Wuhan, China, #15934-1-AP, 1:200), anti-postsynaptic density-95 (PSD95) antibody (Abcam, #ab13552, 1:200), anti-synaptophysin antibody (Abcam, #ab32127, 1:500), anti-phospho-tau (Ser202, Thr205) (Invitrogen, Frederick, MD, #MN1020, 1:200), anti-phospho-tau (Thr231) (Beyotime, Shanghai, China, #AF1951, 1:100), anti-phospho-tau (Ser416) (Abcam, #ab119391, 1:100), anti-GFP (Abcam, #ab1218, 1:1000), anti-dsDNA marker (Santa Cruz Biotechnology, Dallas, TX, #SC-58749, 1:50), and anti-γH2AX (phospho S139) (Abcam, #ab81299, 1:250).

The following secondary antibodies were used for immunohistochemistry and immunocytochemistry in this study: Donkey anti-Mouse IgG H&L (Alexa Fluor® 488) (Abcam, #ab150105, 1:500), Donkey anti-Mouse IgG H&L (Alexa Fluor® 555) (Abcam, #ab150110, 1: 500), Donkey anti-Rabbit IgG H&L (Alexa Fluor® 488) (Abcam, #ab150073, 1: 500), Donkey anti-Rabbit IgG H&L (Alexa Fluor® 647) (Abcam, #ab150063, 1: 500), Donkey anti-Goat IgG H&L (Alexa Fluor® 555) (Abcam, #ab150130, 1: 500), Donkey anti-Chicken IgY (H + L) (Alexa Fluor® 488) (Yeasen, Shanghai, China, #34606ES60, 1: 500), Donkey anti-Chicken IgY (H + L) (Alexa Fluor® 594) (Yeasen, #34612ES60, 1: 500), Goat anti-Mouse IgG (HRP) (Abcam, #ab6789, 1: 300), and Goat anti-Rabbit IgG (HRP) (Abcam, #ab6721, 1: 300).

### RNA extraction and quantitative PCR (qPCR)

Total RNA was extracted from cell lysates using TRIzol reagent. Reverse transcription was performed by EasyQuick RT MasterMix (Cwbio, Taizhou, Jiangsu, China, #CW2019M) according to the manufacturer’s instructions. Relative level of cDNA was determined by real-time qPCR using the 7500 Fast Real-Time PCR System (Applied Biosystems, Foster City, CA) and SYBR Select Master Mix (Applied Biosystems, #4472908). Expression levels of the target genes were normalized to β-actin. The primer sequences used in this study are provided in Additional file 1: Table S2.

### Brain lysate preparation

The mouse hippocampal tissues were homogenized in RIPA lysis buffer (MedChemExpress, #HY-K1001) containing phosphatase inhibitor cocktails (Solarbio Left Sciences, Beijing, China, #P1260) and protease inhibitor cocktail set I (Millipore, #539131) using Tissue LyserII (QIAGEN, Hilden, Germany) and then centrifuged at 15,000 × *g* for 30 min at 4 °C to collect the supernatant (RIPA-soluble fraction). The pellets were resuspended in guanidine buffer (5.0 M guanidine-HCl/50 mM Tris–HCl, pH 8.0) and centrifuged at 15,000× *g* for 1 h at 4 °C to obtain supernatants containing insoluble proteins (RIPA-insoluble fraction). The protein concentrations of soluble and insoluble fractions were determined using the BCA protein assay (Thermo Fisher Scientific, #23225) according to the manufacturer’s instructions.

### Western blotting

Protein samples from mouse hippocampus tissues or cell lysates were separated in 10%–12% SDS-PAGE gels (Invitrogen) and transferred onto nitrocellulose membranes (Merck Millipore). After blocking with 5% non-fat milk for 1 h at room temperature, the membrane was incubated with the corresponding primary antibodies overnight at 4 ℃. Then HRP-conjugated secondary antibodies were applied at a concentration of 1:5000 for 1 h at room temperature. The bands in immunoblots were visualized by enhanced chemiluminescence using an Amersham imager 680 imaging system (GE Healthcare, Pittsburgh, PA) and quantified by densitometry and Image J software.

The following primary antibodies were used for Western blotting: anti-β-actin (Abcam, #ab8226, 1:1000), anti-DNase II (Proteintech, #15934-1-AP, 1:500), anti-phospho-tau (Ser202, Thr205) (Invitrogen, #MN1020, 1:500), anti-phospho-tau (Thr231) (Beyotime, #AF1951, 1:500), anti-phospho-tau (Ser416) (Abcam, #ab119391, 1:1000), anti-CDK5 (Abcam, #ab40773, 1:2000), anti-p35/p25 (Cell Signaling Technology, #2680, 1:1000), anti-PSD95 (Abcam, #ab238135, 1:1000), anti-synaptophysin (Abcam, #ab32127, 1:1000), anti-interferon regulatory factor 3 (IRF3) (Beyotime, #AF2485, 1:1000), anti-phospho-IRF3 (Ser396) (Cell Signaling Technology, #29047, 1:1000), anti-TANK-binding kinase 1 (TBK1) (Abcam, #ab40676, 1:1000), anti-phospho-TBK1/NAK (Ser172) (Cell Signaling Technology, #5483, 1:1000), anti-Bax (Beyotime, #AF1270, 1:1000), anti-caspase3 (Abcam, #ab32351, 1:1000), anti-cleaved-caspase3 (Abcam, #ab32042, 1:1000), anti-Tuj1 (Abcam, #ab18207, 1:1000), anti-MAP2 (Abcam, #ab32454, 1:1000), anti-GFAP (Cell Signaling Technology, #3670S, 1:1000), anti-IBA1 (Abcam, #ab283319, 1:1000), anti-PP2A antibody (Abcam, #ab32104, 1:2000), anti-phospho-PP2A (Tyr307) (Abcam, # ab314196, 1:1000), anti-phospho-GSK-3β (Tyr216) (BD Biosciences, San Jose, CA, #612313, 1:1000), anti-GSK-3β (Cell Signaling Technology, #9315S, 1:1000), anti-Chk1 (Cell Signaling Technology, #2360, 1:1000), anti-phospho-Chk1 (Ser317) (Cell Signaling Technology, #2344, 1:1000), anti-CaMKII-α (Cell Signaling Technology, #50049S, 1:1000), anti-phospho-CaMKII-α (Thr286) (Cell Signaling Technology, #12716 T, 1:1000), anti-Chk2 (Beyotime, #AF2020, 1:1000), anti-phospho-Chk2 (Thr68) (Cell Signaling Technology, #2661, 1:1000), anti-Calpain2 (Abcam, #ab126600, 1:1000), anti-KIAA1524 (CIP2A) (Abclonal, Boston, MA, #A12267, 1:1000), and anti-STING (Beyotime, #AG5348, 1:1000).

The following secondary antibodies were used for Western blotting assay in this study: Goat anti-rabbit IgG (HRP) (Abcam, #ab6721, 1:10,000) and Goat anti-mouse IgG (HRP) (Abcam, #ab6789, 1:10,000).

### Measurement of IL-6, IL-1β and TNF-α

The levels of IL-6, IL-1β, and TNF-α in the brain lysates of mice were determined with corresponding ELISA kits (Biolegend, San Diego, CA, #431304 for IL-6; #432601 for IL-1β; #430904 for TNF-α) according to the manufacturer’s protocols. A SpectraMax M5 microplate reader (Molecular Devices, Sunnyvale, CA) was used to measure the absorbance at 450 nm.

### Tau measurement

The p-tau and total-tau levels in hippocampus of mice were determined using the MULTI-SPOT Phospho (Thr231)/Total tau assay (Meso Scale Diagnostics, Rockville, MD, #K15121D) according to the instructions. Briefly, 25 μl of mouse brain homogenates were added to the plate, and the plate was incubated at room temperature for 1 h with shaking at 500 rpm. After washing for 4 times, the detection antibody was added and the plate was further incubated for 1 h at room temperature. After adding the read buffer, the tau levels were measured by the MSD-S600 reader.

### Statistical analysis

Statistical testing was performed using Prism (GraphPad Software, v8.0.2). For comparisons between groups, first, the Shapiro–Wilk test (Sigma-Plot) was performed to test if the data were normally distributed. For normally distributed data, statistical analysis was performed with one-way or two-way ANOVA followed by Tukey’s test or with unpaired* t*-test with two-tailed *P* values. For non-normally distributed data, Mann–Whitney test (two groups) or Kruskal–Wallis one-way ANOVA on ranks (three or more groups) with Two-stage step-up method Benjamini, Krieger and Yekutieli test was used. Data are expressed as group mean ± SEM, and* P* < 0.05 was considered statistically significant.

## Results

### Neuronal DNase II levels are decreased age-dependently in the brains of WT mice, Tau-P301S mice and AD patients

**Fig. 1:**
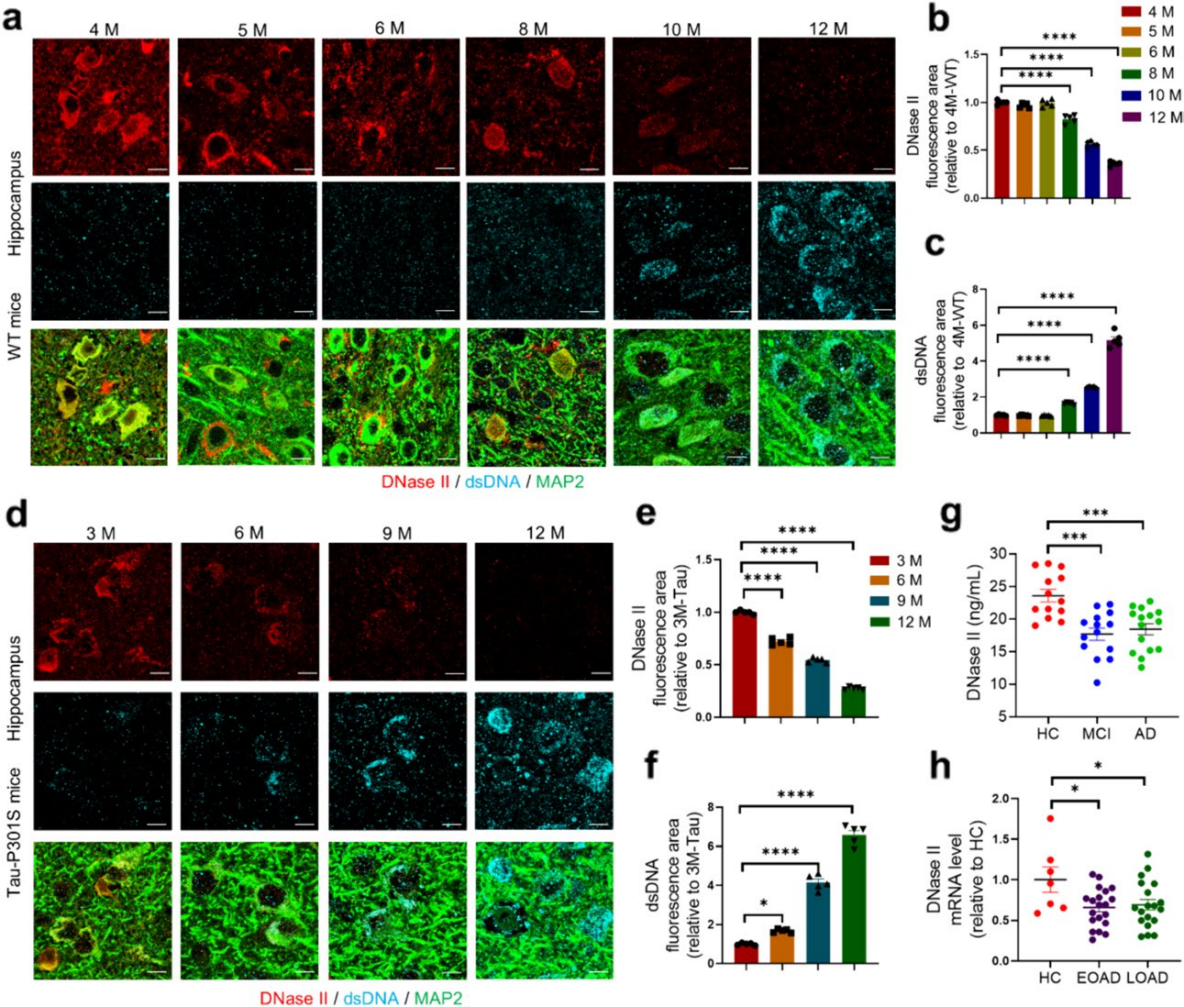
Neuronal DNase II is decreased age-dependently in the hippocampus of WT mice, Tau-P301S mice and AD patients. **a** Immunolabeling of DNase II (red), dsDNA (cyan) and MAP2 (green) in the hippocampus of 4-, 5-, 6-, 8-, 10-, or 12-month-old WT mice. Scale bars, 3 μm. **b**, **c** Quantification of DNase II and dsDNA fluorescent areas in (**a**) by Image J software. *n* = 5 mice per group. **d** Immunolabeling of DNase II (red), dsDNA (cyan) and MAP2 (green) in the hippocampus of 3-, 6-, 9-, and 12-month-old Tau-P301S mice. Scale bars, 3 μm. **e**, **f** Quantification of DNase II and dsDNA fluorescent areas in (**d**) by Image J software. *n* = 5 mice per group. **g** DNase II levels in plasma samples from healthy control (HC), mild cognitive impairment (MCI) patients and AD patients. *n* = 13 to 15 samples per group. **h** Relative mRNA expression of DNase II in the hippocampus of patients with early onset AD cases (EOAD), late onset AD cases (LOAD) and healthy elderly. *n* = 7 to 20 samples per group. Data are mean ± SEM, Mann–Whitney test. Data in **b**, **c**, **e**–**g** were analyzed with one-way ANOVA followed by Tukey’s multiple comparison test. *P < 0.05, ***P < 0.001, ****P < 0.0001

Previous reports have demonstrated that damaged DNA accumulates in neurons of the elderly population and patients with neurological disorders [14, 20]. To explore the underlying mechanism, we identified the levels of DNase II in the hippocampal and cortical neurons of WT and Tau-P301S mice and in the plasma and brains of AD patients. Immunohistochemistry results indicated that the level of neuronal DNase II began to drop at age 8 months and further decreased with age in the hippocampus and cortex of WT mice (Fig. 1a, b and Fig. S1a, b). Correspondingly, dsDNA accumulation increased in the cytoplasm of neurons (Fig. 1a, c and Fig. S1a, c). Moreover, Tau-P301S mice showed age-dependent decrease of DNase II (Fig. 1d, e and Fig. S1d, e) and accumulation of cytoplasmic DNA (Fig. 1d, f and Fig. S1d, f) in hippocampal and cortical neurons at 6, 9 and 12 months. Consistently, Western blotting results confirmed the decrease of DNase II in the brains of WT mice with age (Fig. S1g, h). Besides, the levels of DNase II were also significantly decreased in the brains of Tau-P301S mice at 6, 9, and 12 months of age, compared with the age-matched WT mice (Fig. S1i, j).

In addition, patients with AD and mild cognitive impairment showed remarkably decreased plasma levels of DNase II compared to the healthy controls (Fig. 1g). Moreover, EOAD and LOAD patients exhibited significantly lower mRNA expression of DNase II compared to the healthy elderly people (Fig. 1h).

### DNase II deficiency promotes tau phosphorylation in primary neurons

**Fig. 2:**
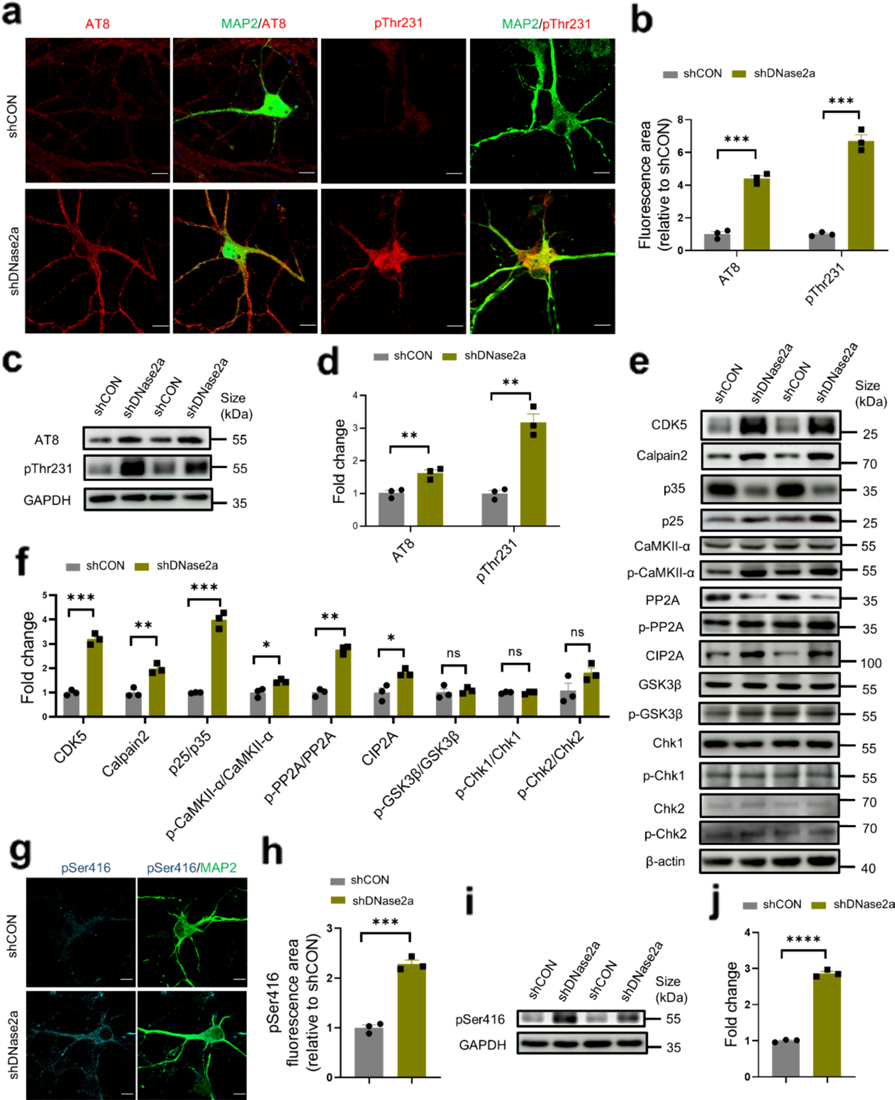
DNase II deficiency promotes tau phosphorylation in primary hippocampal neurons through activation of the CDK5, CaMKII and PP2A signaling pathway. **a** Representative images of AT8 (red) and pThr231 (red) fluorescence staining in primary hippocampal neurons infected with shDNase2a or shCON. Scale bars, 10 μm. **b** Quantification of AT8 and pThr231 fluorescent areas in (**a**) by Image J software. **c** Western blotting of AT8 and pThr231 in the primary hippocampal neurons infected with shDNase2a or shCON. **d** Quantitation of the levels of AT8 and pThr231 in (**c**). **e** Western blotting of CDK5, Calpain2, p25, p35, CaMKII-α, p-CaMKII-α, PP2A, p-PP2A, CIP2A, GSK-3β, p-GSK-3β, Chk1, p-Chk1, Chk2 and p-Chk2 in the primary hippocampal neurons infected with shDNase2a or shCON. **f** Quantitation of the bands in (**e**) by the Image J software. **g** Representative images of pSer416 (cyan) fluorescence staining in primary hippocampal neurons infected with shDNase2a or shCON. Scale bars, 10 μm. **h** Quantification of pSer416 fluorescent area in (**g**) by the Image J software. **i** Western blotting of pSer416 in the primary hippocampal neurons infected with shDNase2a or shCON. **j** Quantitation of the levels of pSer416 in (**i**). In **a**, **c**, **e**, **g** and **i**, data are representative of three independent experiments. In **b**, **d**, **f**, **h**, and **j**, data were pooled from three independent experiments. Mean ± SEM, unpaired* t*-test with two-tailed *P* values was used for statistical analysis. *P < 0.05, **P < 0.01, ***P < 0.001, ****P < 0.0001, ns, not significant

To investigate whether DNase II deficiency is involved in the pathogenesis of tauopathies, we constructed a lentivirus system carrying *Dnase2a* shRNA (shDNase2a) to knockdown DNase II in primary hippocampal neurons (Fig. S2a–d). Immunocytochemistry and Western blotting consistently showed that DNase II deficiency induced significant increases in the levels of p-tau phosphorylated at Ser202/Thr205 and Thr231 in primary hippocampal neurons (Fig. 2a–d).

### CDK5, CaMKII and PP2A are involved in tau phosphorylation induced by neuronal DNase II deficiency

Multiple protein kinases and phosphatases, such as GSK-3β, CDK5, Chk1, Chk2 and CaMKII, are responsible for tau phosphorylation [21, 22, 25]. We found significantly increased levels of CDK5 as well as kinase activities of CDK5 (measured by the p25/p35 ratio [31, 32]), CaMKII-α (measured by p-CaMKII-α/CaMKII-α ratio) rather than GSK-3β, Chk1 and Chk2 kinase activity in neurons infected with shDNase2a (Fig. 2e, f). Previous reports demonstrated that Calpain2 can be activated under neurotoxicity such as DNA damage to cleave p35 to produce p25 [33], which in turn activates CDK5 and promotes tau phosphorylation [34]. Correspondingly, the levels of p25/p35 and Calpain2 were remarkably increased in DNase II-deficient neurons (Fig. 2e, f). To further verify the effect of CDK5 on the tau phosphorylation induced by DNase II knockdown, we added compound roscovitine, an inhibitor of CDK5 [28], to primary hippocampal neurons with or without DNase II knockdown. Roscovitine significantly inhibited tau phosphorylation at Ser202/Thr205 and Thr231 in DNase II-deficient neurons (Fig. S2e–i). As tau phosphorylation is also regulated by phosphatases such as PP2A, the activity of PP2A was also assessed. We found increased levels of the inactive phosphatase PP2A (measured by p-PP2A/PP2A) in DNase II-deficient neurons. The phosphorylated form of PP2A (p-PP2A), which downregulates the activity of PP2A [35], was significantly elevated (Fig. 2e, f). Moreover, tau hyperphosphorylation at Ser202/Thr205 and pThr231 was obviously inhibited by DT061 [29], an activator of PP2A (Fig. S2j–n). Furthermore, cancerous inhibitor of protein phosphatase 2A (CIP2A), an endogenous PP2A inhibitor [36] that is overexpressed in AD brain [37], was significantly increased in DNase II-deficient neurons (Fig. 2e, f). Moreover, active CaMKII-α was increased (Fig. 2e, f), which may explain the increased levels of tau phosphorylated at serine 416 (a CaMKII-α target site) in DNase II-deficient primary hippocampal neurons (Fig. 2g–j).

### DNase II downregulation induces neuronal apoptosis via the cGAS–STING pathway

Downregulation of DNase II in peripheral leukocytes leads to apoptosis through aberrant activation of the cGAS**–**STING pathway via cytoplasmic accumulation of nuclear DNA [38]. We here found that the immunoreactivity of γH2AX, a marker of double-stranded DNA breaks, was significantly increased in the nuclear and the cytoplasm of DNase II-deficient primary hippocampal neurons (Fig. S3a, b). Next, we measured the levels of STING and downstream mediators of the cGAS**–**STING cytoplasmic DNA sensing pathway, TBK1, phosphorylated TBK1 (p-TBK1), IRF3 and phosphorylated IRF3 (p-IRF3). Downregulation of DNase II remarkably upregulated the levels of STING, p-TBK1/TBK1 and p-IRF3/IRF3 in DNase II-deficient neurons (Fig. S3c, d), suggesting activation of the cGAS**–**STING signaling pathway. Consistent with previous report in leukocytes, knockdown of DNase II induced apoptosis of primary hippocampal neurons as detected by TUNEL staining (Fig. S3e, f). Correspondingly, apoptosis-related proteins, such as Bax and cleaved-caspase3 (Cleaved-cas3), were upregulated in DNase II-deficient primary hippocampal neurons, while the level of caspase-3 remained unchanged (Fig. S3c, d).

A previous study showed that activation of cGAS-STING can lead to activation of the IFN-I signaling pathway [6]. We here found that the mRNA expression of IFN-I-related genes *Irf7*, *Ifit1*, *Ifitm3*, *Igtp* and *Isg15* was significantly increased in the primary hippocampal neurons infected with shDNase2a other than shCON (Fig. S3g). These findings demonstrated that DNase II deficiency activates the cGAS–STING and IFN-I signaling pathways, and induces neuronal apoptosis (Fig. S3h).

### Neuronal DNase II deficiency promotes tau phosphorylation and aggregation in WT mice

AAV-shDNase2a or AAV-CON vector, which was controlled by a neuronal specific promoter hSyn, was injected into the cornu ammonis 1 (CA1) and the dentate gyrus (DG) of dorsal hippocampi of 3-month-old C57BL/6 mice (Fig. S4a, b). Four weeks later, the AAV vector-related GFP fluorescence was present throughout the dorsal hippocampus, specifically colocalized with MAP2^+^ neurons but not with microglia or astrocytes, showing the neuronal-specific expression of AAV (Fig. S4c–e).

**Fig. 3:**
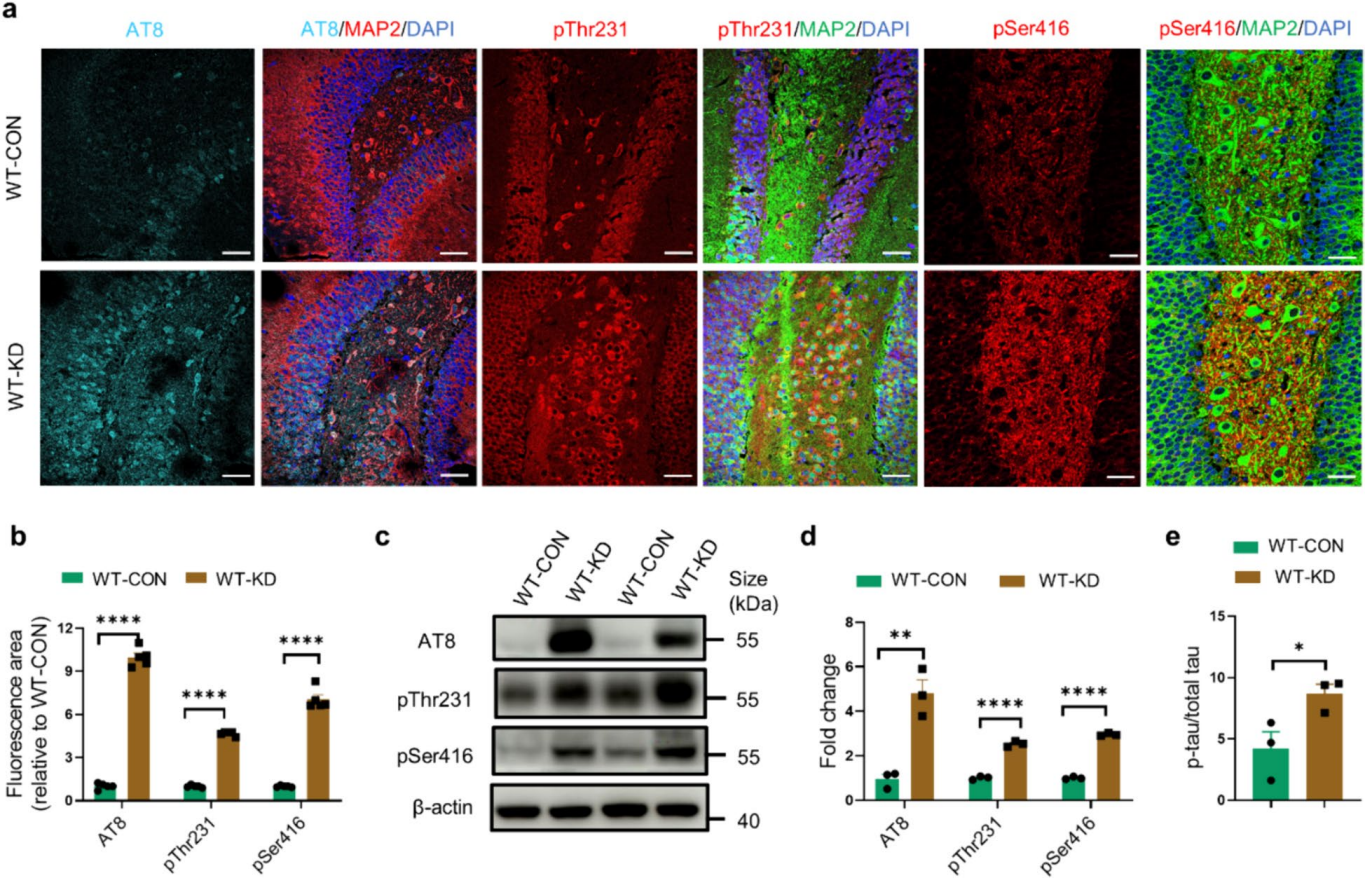
DNase II deficiency promotes tau phosphorylation in vivo. **a** Representative images of AT8 (cyan), pThr231 (red) and pSer416 (red) fluorescence staining in the hippocampal DG region of WT-CON mice and WT-KD mice. Scale bars, 50 μm. **b** Quantification of AT8, pThr231 and pSer416 fluorescent areas in (**a**). *n* = 5 mice per group. **c** Western blotting of AT8, pThr231 and pSer416 in the hippocampal homogenates of WT-CON mice and WT-KD mice. **d** Quantitation of the levels of AT8, pThr231 and pSer416 in (**c**). **e** The p-tau/total tau ratio in the hippocampal homogenates of WT-CON mice and WT-KD mice measured by electrochemiluminescence assay. In **c**, data are representative of three independent experiments. In **d** and **e**, data were pooled from three independent experiments. Data are presented as mean ± SEM of three independent experiments, and unpaired* t*-test with two-tailed *P* values was used for statistical analysis. *P < 0.05, **P < 0.01, ****P < 0.0001

Consistent with the in vitro results, the levels of tau phosphorylated at Ser202/Thr205, Thr231 and Ser416 in hippocampus were all significantly increased in the DNase II-deficient WT (WT-KD) mice compared to WT control (WT-CON) mice (Fig. 3a, b). Western blotting analysis also showed increased levels of phosphorylated tau in hippocampal lysates of WT-KD mice (Fig. 3c, d). These results were further confirmed by the pThr231/total tau ratio in hippocampal lysates by electrochemiluminescence assay (Fig. 3e). Correspondingly, the level of CDK5 and the kinase activities of CDK5 (p25/p35), active CaMKII-α (p-CaMKII-α/CaMKII-α), inactive phosphatase PP2A (p-PP2A/PP2A) and CIP2A, rather than GSK-3β, Chk1 and Chk2 kinase activity, were significantly increased in WT-KD relative to WT-CON mice (Fig. S4f, g).

Abnormally phosphorylated tau loses its microtubule-binding function, and aggregates to form oligomers or larger aggregates, leading to synaptic loss and cognitive deficits [39]. We here found that the levels of SDS-resistant tau aggregates in both RIPA-soluble and RIPA-insoluble fractions were significantly increased in WT-KD mice compared to WT-CON mice (Fig. S4h–j).

### Neuronal DNase II deficiency induces cognitive impairment and synaptic loss in WT mice

**Fig. 4:**
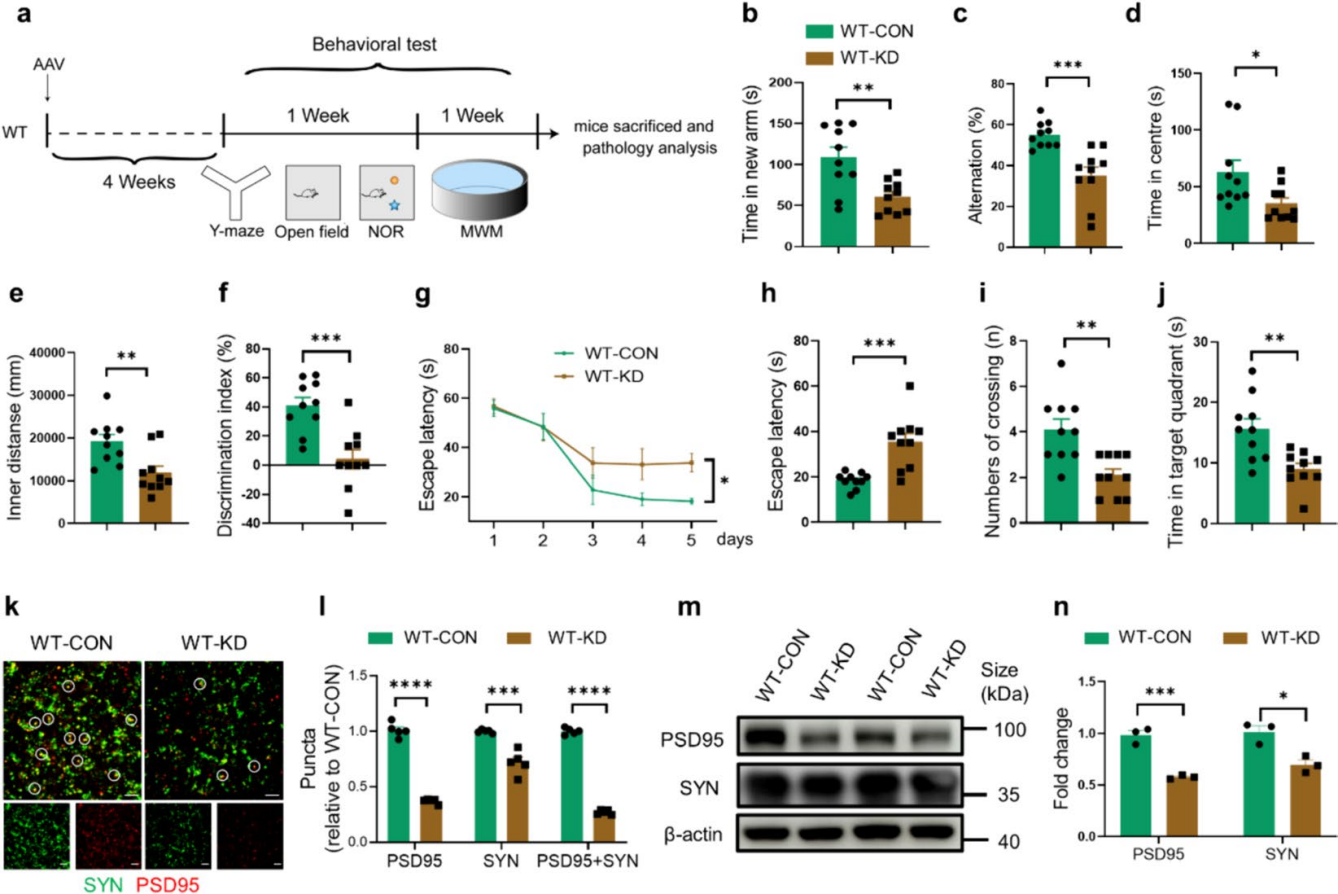
Neuronal DNase II deficiency induces cognitive impairment and synaptic loss in WT mice. **a** Schematic representation of the pharmacological treatment and experimental measurement. **b** The time spent in the novel arm in the forced Y-maze test. **c** The alternation in a spontaneous Y-maze. **d**, **e** The time spent and distances traveled in the central area of the open field. **f** Discrimination index in the novel object recognition test. **g** The latency to find the hidden platform during training trials of MWM test. Data are mean ± SEM, two-way ANOVA followed by Tukey’s multiple comparison test. **h** The latency to the position of the removed platform during probe trials of MWM test. **i** The number of platform crossings during probe trials of the MWM test. Mann–Whitney test. **j** The time spent in the target quadrant during probe trials of MWM test. **k** Immunolabeling of PSD95 (red) and synaptophysin (SYN) (green) puncta in the brains of WT-CON mice and WT-KD mice. Circles indicate co-localization of PSD95 and SYN puncta. Scale bars, 5 μm. **l** Quantification of synaptic puncta or their apposition. *n* = 5 mice per group. **m** Western blotting of PSD95 and SYN in the hippocampal homogenates of WT-CON mice and WT-KD mice. **n** Quantitation of the levels of PSD95 and SYN in the brains of mice. In **b**–**j**, *n* = 10 mice per group. In **m**, data are representative of three independent experiments. In **n**, data are pooled from three independent experiments. In **b**-**f**, **h**, **j**, **l** and **n**, data are presented as mean ± SEM and analyzed with unpaired* t*-test with two-tailed *P* values. *P < 0.05, **P < 0.01, ***P < 0.001, ****P < 0.0001

A series of behavioral tests were performed to assess the effect of DNase II knockdown on the cognition of WT mice (Fig. 4a). No significant differences in mouse survival or motor function was observed in any experimental groups of 4-month-old WT mice (Fig. S5a-d). In forced Y-maze test, WT-KD mice showed reduced time spent in the novel arm of Y-maze (Fig. 4b, Fig. S5e), as well as less alternation in the spontaneous Y-maze test compared with the WT-CON mice (Fig. 4c). In the open field test, WT-KD mice spent less time (Fig. 4d) and traveled less distances (Fig. 4e) in the central area (Fig. S5f). In the NOR test, WT-KD mice showed decreased discrimination index when compared with the WT-CON mice (Fig. 4f). In the MWM training test, the WT-KD mice showed impaired spatial learning ability with longer latency than the WT-CON mice (Fig. 4g). In the probing test of MWM, WT-KD mice showed increased escape latency (Fig. 4h), reduced platform crossings (Fig. 4i), and shorter time spent in the target quadrant (Fig. 4j, Fig. S5g) compared with the WT-CON mice. These results indicated that DNase II knockdown induced cognitive deficits in 4-month-old WT mice.

We also determined the levels of neuronal DNase II and dsDNA in the cytoplasm of neurons in 4-month-old WT mice. Compared with WT-CON mice, the levels of neuronal DNase II were significantly decreased, and resulted in increased dsDNA accumulation in the brains of WT-KD mice (Fig. S5h, i).

Next, we determined the effect of DNase II deficiency on synapses. The levels of PSD95 and synaptophysin (SYN), and the number of intact synapses (indicated by co-localization of PSD95 and SYN) were significantly decreased in the brains of WT-KD mice compared to WT-CON mice (Fig. 4k, l). These findings were confirmed by Western blotting analysis of PSD95 and SYN in the hippocampus lysates (Fig. 4m, n). Furthermore, significantly increased astrogliosis and microgliosis were observed in the brains of WT-KD mice (Fig. S5j, k). Western blotting analysis consistently showed that the levels of GFAP and IBA1 were increased in the hippocampal lysates of WT-KD mice compared to WT-CON mice (Fig. S5l, m). ELISA results showed higher levels of inflammatory cytokines IL-6, IL-1β and TNF-α in WT-KD mice than in WT-CON mice (Fig. S5n). Moreover, TUNEL staining showed abundant TUNEL-positive cells in the hippocampus of WT-KD mice relative to WT-CON mice (Fig. S5o, p).

### Neuronal DNase II deficiency exacerbates cognitive deficits and tauopathy in Tau-P301S mice

**Fig. 5:**
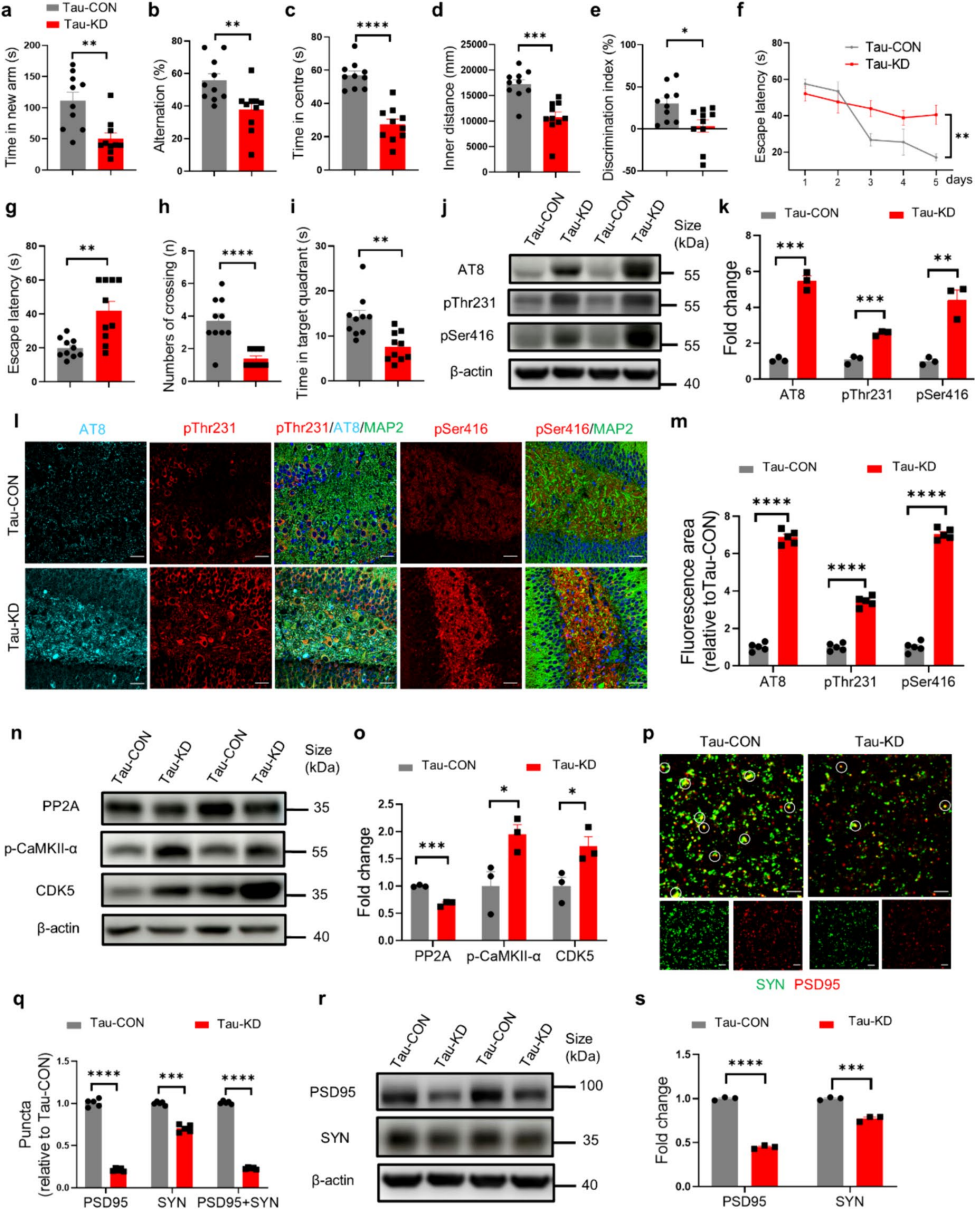
Neuronal DNase II deficiency induces tau phosphorylation and exacerbates cognitive impairment and synaptic loss in Tau-P301S mice. **a** The time spent in the novel arm in forced Y-maze test. **b** Alternation in the spontaneous Y-maze test. **c**, **d** The time spent and distance traveled in the central area of the open field. **e** Discrimination index in the novel object recognition test. **f** The latency to find the hidden platform during training trials of MWM test. *n* = 10 mice per group. **g** The latency to the position of the removed platform during probe trials of the MWM test. **h** The number of platform crossings during probe trials of the MWM test. Mann–Whitney test. **i** The time spent in the target quadrant during probe trials of MWM test. **j** Western blotting of AT8, pThr231 and pSer416 in the hippocampal homogenates of Tau-CON mice and Tau-KD mice. **k** Quantitation of the levels of AT8, pThr231 and pSer416 in (**j**). **l** Immunolabeling of AT8 (cyan), pThr231 (red) and pSer416 (red) in the hippocampal DG region of Tau-CON mice and Tau-KD mice. Scale bars, 50 μm. **m** Quantification of AT8, pThr231 and pSer416 fluorescent areas in (**l**). *n* = 5 mice per group. **n** Western blotting of PP2A, p-CaMKII-α and CDK5 in the hippocampus lysates of Tau-CON mice and Tau-KD mice. **o** Quantitation of the levels of PP2A, p-CaMKII-α and CDK5 in (**n**). **p** Immunolabeling of PSD95 (red) and synaptophysin (SYN) (green) puncta in the brains of Tau-CON mice and Tau-KD mice. Circles indicate co-localization of PSD95 and SYN puncta. Scale bars, 5 μm. **q** Quantification of synaptic puncta or their apposition in (**p**). *n* = 5 mice per group. **r** Western blotting of PSD95 and SYN in the hippocampal homogenates of Tau-CON mice and Tau-KD mice. **s** Quantitation of the levels of PSD95 and SYN in the brains of mice in (**r**). In **a–i**, *n* = 10 mice per group. In **j**, **n** and **r**, data are representative of three independent experiments. In **k**, **o** and **s**, data are pooled from three independent experiments. Data are presented as mean ± SEM, and analyzed with unpaired* t*-test with two-tailed *P* values (**a**–**e**, **g**, **i**, **k**, **m**, **o**, **q** and **s**) or two-way ANOVA followed by Tukey’s multiple comparison test (**f**). *P < 0.05, **P < 0.01, ***P < 0.001, ****P < 0.0001

To further assess the effect of DNase II deficiency on the neuropathology and cognition in tau mouse model, AAV-shDNase2a or AAV-shCON was bilaterally injected into the hippocampus of 3-month-old Tau-P301S mice. At 4 weeks after injection, no significant differences in mouse survival or motor function were observed between groups (Fig. S6a–d). The results of forced Y-maze, spontaneous Y-maze, open filed, NOR and MWM tests demonstrated that DNase II knockdown aggravated cognitive deficits in 4-month-old Tau-P301S mice (Fig. 5a–i, Fig. S6e–g).

As expected, the levels of neuronal DNase II were significantly decreased and dsDNA accumulation increased in the brains of DNase II-deficient Tau-P301S (Tau-KD) mice compared with Tau-P301S control (Tau-CON) mice (Fig. S6h, i). Correspondingly, the levels of phosphorylated tau were significantly increased in Tau-KD mice (Fig. 5j–m). The levels of CDK5 and p-CaMKII-α were increased, while the level of PP2A was reduced in Tau-KD mice (Fig. 5n, o). Moreover, significant decreases in the levels of PSD95 and SYN as well as the number of intact synapses were observed in Tau-KD mice, as compared to the Tau-CON mice (Fig. 5p–s).

Furthermore, DNase II knockdown induced astrogliosis and microgliosis (Fig. S6j–m) and increased the levels of inflammatory cytokines IL-6, IL-1β and TNF-α (Fig. S6n). More apoptotic cells were detected in the brains of Tau-KD mice (Fig. S6o, p).

### Neuronal DNase II overexpression ameliorates cognitive deficits and tauopathies in Tau-P301S mice

**Fig. 6:**
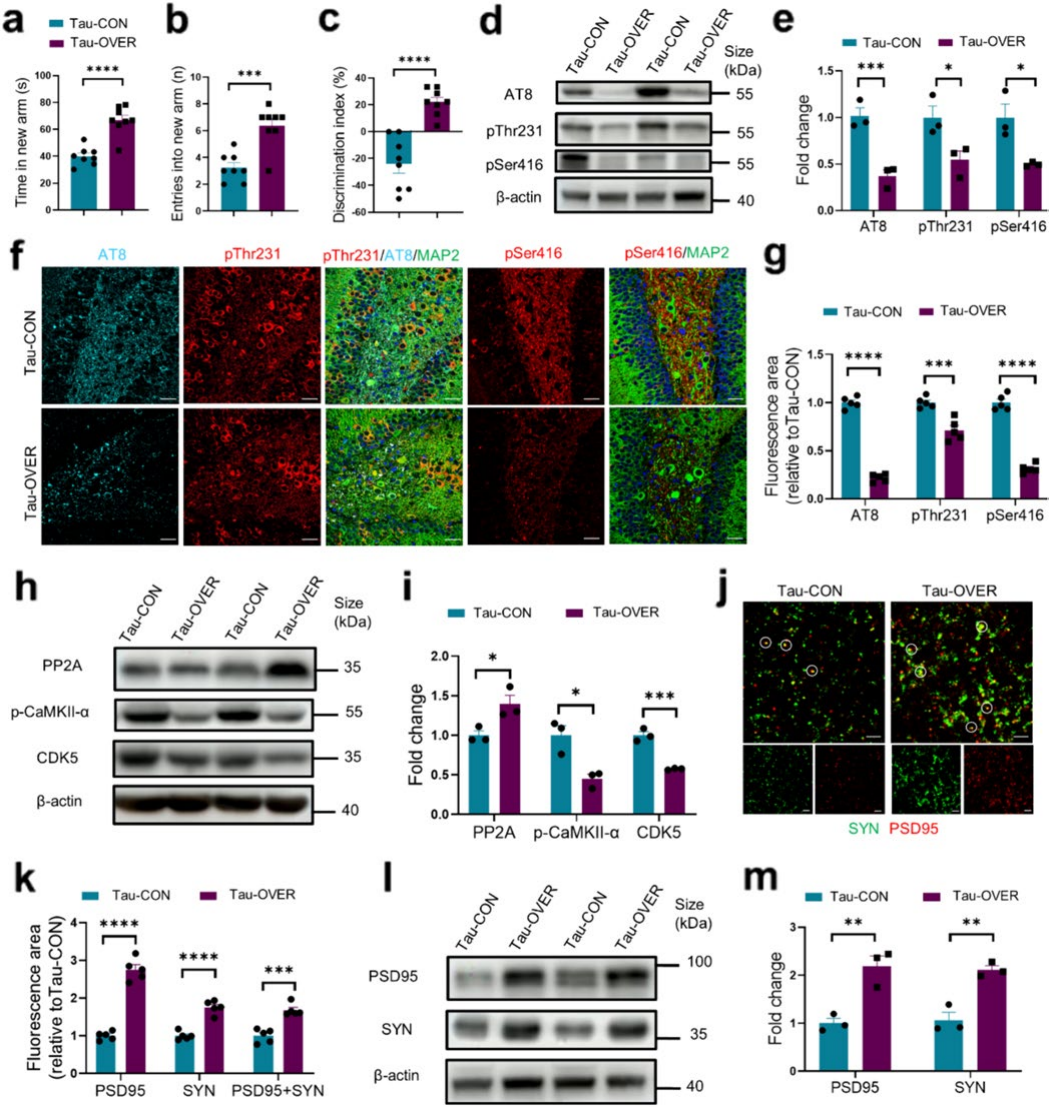
Neuronal DNase II overexpression prevents cognitive deficits and tau phosphorylation in Tau-P301S mice. **a**, **b** The time spent and entries in the new arm in forced Y-maze. *n* = 8 mice per group. **c** Discrimination index in the novel object recognition test. *n* = 8 mice per group. **d** Western blotting of AT8, pThr231 and pSer416 in the hippocampal homogenates of Tau-CON mice and Tau-OVER mice. **e** Quantitation of the levels of AT8, pThr231 and pSer416 in (**d**). **f** Representative images of AT8 (cyan), pThr231 (red) and pSer416 (red) fluorescence staining in the hippocampal DG region of Tau-CON mice and Tau-OVER mice. Scale bars, 50 μm. **g** Quantification of AT8, pThr231 and pSer416 fluorescent areas in (**f**). *n* = 5 mice per group. **h** Western blotting of PP2A, p-CaMKII-α and CDK5 in the hippocampal homogenates of Tau-CON mice and Tau-OVER mice. **i** Quantitation of the levels of PP2A, p-CaMKII-α and CDK5 in (**h**). **j** Immunolabeling of PSD95 (red) and synaptophysin (SYN) (green) puncta in the brains of Tau-CON mice and Tau-OVER mice. Circles indicate Co-localization of PSD95 and SYN puncta. Scale bars, 5 μm. **k** Quantification of synaptic puncta or their apposition in (**j**). *n* = 5 mice per group. **l** Western blotting of PSD95 and SYN in the hippocampal homogenates of Tau-CON mice and Tau-OVER mice. **m** Quantitation of the levels of PSD95 and SYN in the brains of mice in (**l**). In **d**, **h** and **l**, data are representative of three independent experiments. In **e**, **i** and **m**, data are pooled from three independent experiments. Data are presented as mean ± SEM, and analyzed with unpaired* t*-test with two-tailed *P* values (**a**, **c**, **e**, **g**, **i**, **k**, **m**) or Mann–Whitney test (**b**). *P < 0.05, **P < 0.01, ***P < 0.001, ****P < 0.0001

To assess the effect of DNase II overexpression in neurons on the cognitive deficits and tauopathies in Tau-P301S mice, we overexpressed DNase II in neurons of 9-month-old Tau-P301S mice by injecting AAV-DNase2a into the CA1 and DG regions of dorsal hippocampi. Four weeks later, the 10-month-old Tau-P301S mice with neuronal DNase II-overexpression (Tau-OVER) spent more time in the new arm and made more entries into the new arm in the forced Y-maze test (Fig. 6a, b). They also exhibited a marked increase in the discrimination index of the novel object in the NOR test compared to the 10-month-old Tau-P301S control (Tau-CON) mice (Fig. 6c). These results indicated that neuronal DNase II overexpression significantly attenuated the cognitive and memory impairment in aged Tau-P301S mice.

As expected, the levels of neuronal DNase II were significantly increased and dsDNA accumulation decreased in the brains of Tau-OVER mice compared to the Tau-CON mice (Fig. S7a, b). We next assessed the effect of DNase II overexpression on the levels of phosphorylated tau in the hippocampus of the Tau-P301S mice. Western blotting and immunohistochemistry results demonstrated that DNase II overexpression greatly reduced the levels of p-tau in Tau-OVER mice compared to Tau-CON mice (Fig. 6d–g). The levels of CDK5 and p-CaMKII-α were significantly decreased while the level of PP2A was significantly increased with DNase II overexpression in mice, leading to lower levels of tau phosphorylation (Fig. 6h, i).

In addition, the levels of PSD95 and SYN were significantly enhanced (Fig. 6j–m), while astrogliosis, microgliosis and inflammatory cytokines including IL-6, IL-1β and TNF-α were remarkably decreased in the Tau-OVER mice compared to Tau-CON mice (Fig. S7c–g). Moreover, TUNEL staining revealed that DNase II overexpression significantly decreased the number of TUNEL-positive cells (Fig. S7h, i). Thus, DNase II overexpression ameliorates cognitive deficits and tauopathies in Tau-P301S mice.

## Discussion

DNase II has the ability to hydrolyze dsDNA in lysosomes, and its downregulation triggers the cytoplasmic accumulation of nuclear DNA in senescent cells [7]. Given that dsDNA damage is prevalent in the brain and is strongly associated with neuronal health [15, 16, 18, 19], we speculate a role of DNase II deficiency and cytoplasmic dsDNA accumulation in neurodegeneration. In the present study, we showed that the level of neuronal DNase II is downregulated in the brains of aged WT mice, tauopathy mouse model and AD patients, resulting in accumulation of damaged DNA in the cytoplasm, and further tau pathogenesis and cognitive impairment.

Tau phosphorylation is catalyzed by multiple protein kinases and balanced by phosphatases [21, 22, 40]. The regulation of these enzymes by different processes results in tau phosphorylation at different sites [41, 42]. In AD, several kinases such as CaMKII, CDK5, Chk1 and GSK-3β, and phosphatases such as PP2A, play key roles in tau phosphorylation [43]. Here we found that the kinase activities of CDK5 and CaMKII were significantly increased in the DNase II-deficient neurons, while the kinase activities of GSK-3β, Chk1 and Chk2 remained unchanged. Moreover, the inactive phosphatase PP2A and its negative regulator CIP2A were increased in both in vitro or in vivo experiments. Neuronal DNase II deficiency and accumulation of damaged DNA in cytoplasm led to changes in the levels of corresponding enzymes and induced tau phosphorylation on multiple epitopes.

The presence of phosphorylated tau at Ser202/Thr205 in certain temporal and spatial patterns in the hippocampus and cortical brain regions is correlated with Braak stage [42]. Tau phosphorylated at threonine 231 (p-tau231) in cerebrospinal fluid and plasma is a biomarker for AD pathophysiology. P-Tau231 is increased more prominently in preclinical AD and is related to decreased declarative memory and medial temporal atrophy [44, 45]. Consistently, phosphorylation of tau induced by DNase II deficiency and cytoplasmic DNA accumulation caused neuron dysfunction and loss, and cognitive impairment. In contrast, neuronal DNase II overexpression effectively rescued the tauopathies and cognitive deficits in aged Tau-P301S mice.

Hyperphosphorylated tau readily aggregates into neurotoxic oligomers and fibrils [46]. Consistently, here we found higher levels of aggregates of phosphorylated tau in the brains of mice with DNase II knockdown. It has been reported that decreases of DNase II in leukocytes lead to chronic activation of IFN-I signaling mediated by the cGAS–STING pathway [6], and the cGAS–STING pathway initiation also induces cell apoptosis in peripheral senescent cells [47]. We here observed significantly increased expression of IFN-I pathway-related transcripts in the DNase II-deficient neurons. In addition, downregulation of DNase II induced neuronal apoptosis via activating the cGAS–STING signaling pathway. Previous studies have shown that dsDNA burdens in neurons incite neuroinflammation associated with neurodegeneration [48]. Consistently, we also found significant increases in microgliosis, astrogliosis and inflammatory levels in the brains of WT-KD and Tau-KD mice.

## Conclusions

Our findings demonstrated that the DNase II levels were decreased in the brains of aged WT mice, Tau-P301S mice and AD patients, resulting in cytoplasmic accumulation of DNA. DNase II deficiency induces tau phosphorylation by increasing CDK5 and CaMKII and decreasing PP2A in vitro and in vivo, especially in WT mice. The aberrantly phosphorylated tau readily forms aggregates. The later, together with the activation of the cGAS–STING signaling pathway by accumulated DNA, causes neuron dysfunction and loss, neuroinflammation and cognitive deficits. These findings reveal that DNase II deficiency is an early event rather than merely a secondary phenomenon in the pathogenesis of tau-associated neurodegeneration. Therefore, DNase II and cytoplasmic DNA may be potential therapeutic targets for tau-associated disorders.

### Supplementary Information

**Additional file 1: Fig. S1**. Neuronal DNase II is decreased age-dependently in the cortex of WT mice and Tau-P301S mice. **Fig. S2**. DNase II deficiency induces tau phosphorylation by regulating kinases and phosphatases of tau in vitro. **Fig. S3**. DNase II downregulation induces neuronal apoptosis via cGAS–STING pathway. **Fig. S4**. DNase II deficiency increases tau phosphorylation and aggregation by regulating kinases and phosphatases of tau in vivo. **Fig. S5**. Neuronal DNase II deficiency enhances neuroinflammation and apoptosis in 4-month-old WT mice. **Fig. S6**. Neuronal DNase II deficiency exacerbates gliosis and apoptosis in 4-month-old Tau-P301S mice. **Fig. S7**. Neuronal DNase II overexpression reduces neuroinflammation and apoptosis in Tau-P301S mice. **Table S1**. Demographic details of the plasma used in this study. **Table S2**. Primer sequences.

## Data Availability

All data generated and/or analyzed during this study are either included in this article or are available from the corresponding author on reasonable request. This study did not generate new unique reagents.

## Abbreviations

AD: Alzheimer’s disease
CaMKII: Calcium/calmodulin activated protein kinase II
CDK5: Cyclin-dependent-like kinase-5
cGAS: Cyclic GMP-AMP synthase
Chk1: Cell cycle checkpoint kinases1
Chk2: Cell cycle checkpoint kinases2
CIP2A: Cancerous inhibitor of protein phosphatase 2A
DAB: Diaminobenzidine
DNase II: Deoxyribonuclease 2
EOAD: Early onset AD cases
GFAP: Glial fibrillary acidic protein
GSK-3β: Glycogen synthase kinase-3β
IBA1: Ionized calcium-binding adapter molecule 1
IL-1β: Interleukin-1β
IL-6: Interleukin 6
IRF3: Interferon regulatory factor 3
LOAD: Late onset AD cases
MAP2: Microtubule associated protein 2
MCI: Mild cognitive impairment patients
MWM: Morris water maze
NOR: Novel objection recognition
PP2A: Protein phosphatase 2A
PSD95: Postsynaptic density protein-95
STING: Stimulator of interferon genes protein
SYN: Synaptophysin
TBK1: TANK-binding kinase 1
TNF-α: Tumor necrosis factor α
TUNEL: Terminal deoxynucleotidyl transferase mediated dUTP nick-end labeling
IFN-I: Type-I-interferon
