# Supplementary material for: Neuronal double-stranded DNA accumulation induced by DNase II deficiency drives tau phosphorylation and neurodegeneration
Source: Transl Neurodegener. 2024 Aug 2;13:39. doi: 10.1186/s40035-024-00427-8 (PMC11295666; doi:10.1186/s40035-024-00427-8)
Supplement: Supplementary file 1 — Additional file 1: Fig. S1. Neuronal DNase II is decreased age-dependently in the cortex of WT mice and Tau-P301S mice. Fig. S2. DNase II deficiency induces tau phosphorylation by regulating kinases and phosphatases of tau in vitro. Fig. S3. DNase II downregulation induces neuronal apoptosis via cGAS–STING pathway. Fig. S4. DNase II deficiency increases tau phosphorylation and aggregation by regulating kinases and phosphatases of tau in vivo. Fig. S5. Neuronal DNase II deficiency enhances neuroinflammation and apoptosis in 4-month-old WT mice. Fig. S6. Neuronal DNase II deficiency exacerbates gliosis and apoptosis in 4-month-old Tau-P301S mice. Fig. S7. Neuronal DNase II overexpression reduces neuroinflammation and apoptosis in Tau-P301S mice. Table S1. Demographic details of the plasma used in this study. Table S2. Primer sequences. [file 40035_2024_427_MOESM1_ESM.docx]

**Supplementary Information**

**
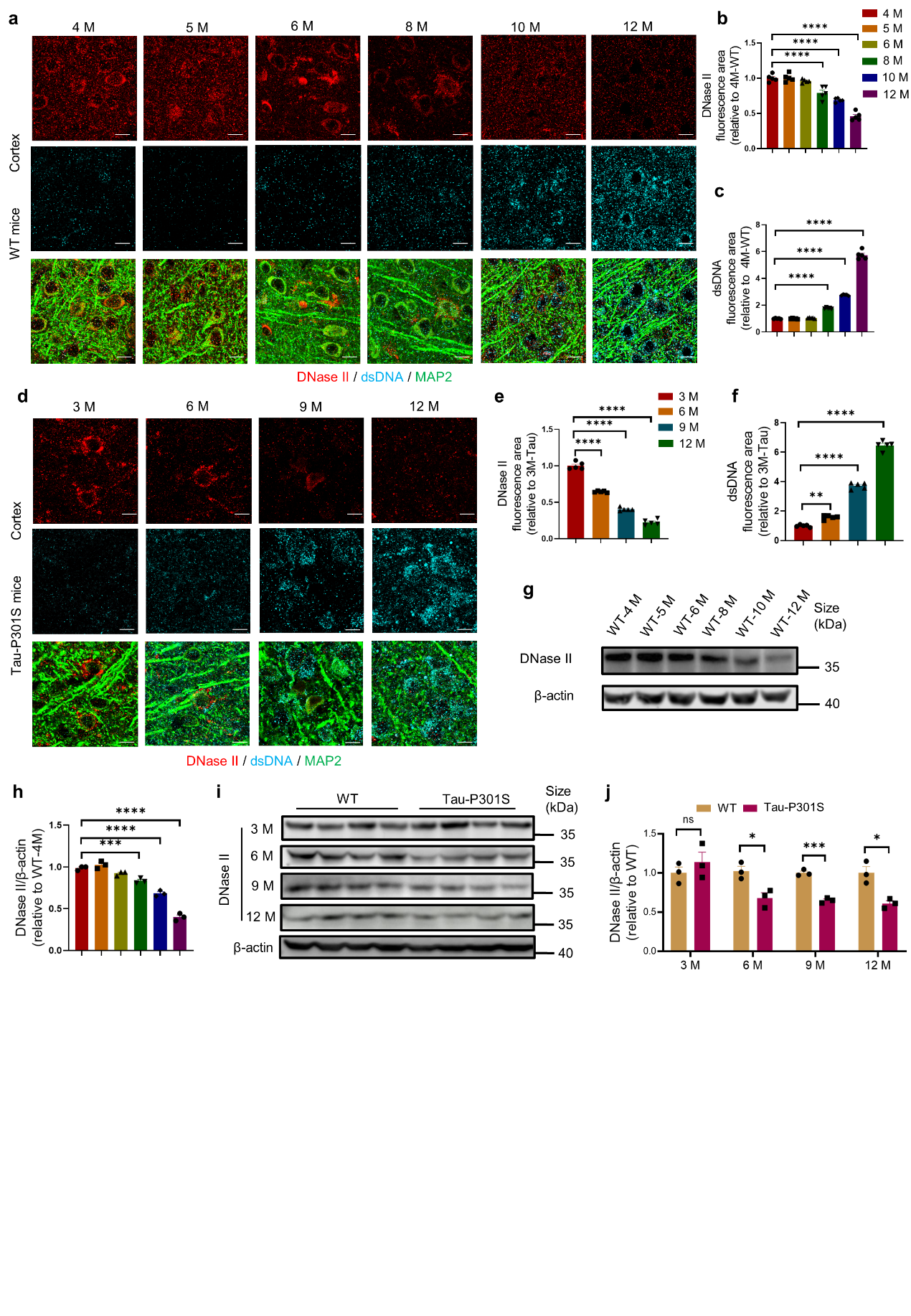
**

**Fig. S1** Neuronal DNase Ⅱ is decreased age-dependently in the cortex of WT mice and Tau-P301S mice. **a** Immunolabeling of DNase Ⅱ (red), dsDNA (cyan) and MAP2 (green) in the cortex of 4-, 5-, 6-, 8-, 10-, and 12-month-old WT mice. Scale bars, 3 μm. **b, c** Quantification of DNase Ⅱ and dsDNA fluorescent area by Image J software in (**a**). *n* = 5 mice per group. **d** Immunolabeling of DNase Ⅱ (red), dsDNA (cyan) and MAP2 (green) in the cortex of 3-, 6-, 9-, and 12-month-old Tau-P301S mice. Scale bars, 3 μm. **e, f** Quantification of DNase Ⅱ and dsDNA fluorescent area by Image J software in (**d**). *n* = 5 mice per group. **g** Western blotting of DNase Ⅱ levels of the brain homogenates of 4-, 5-, 6-, 8-, 10-, and 12-month-old WT mice. **h** Quantitation of DNase Ⅱ levels in (**g**). **i** Western blotting of DNase Ⅱ levels in the brain homogenates of 3-, 6-, 9-, and 12-month-old WT mice and Tau-P301S mice. **j** Quantitation of DNase Ⅱ levels in (**i**), data are mean ± SEM, and unpaired *t*-test with two-tailed *P* values was used for statistical analysis. In **g** and **i**, data are representative of three independent experiments, and in **h** and **j** data are pooled from three independent experiments. In **b**, **c**, **e**, **f** and **h**, data are mean ± SEM, and a one-way ANOVA followed by Tukey’s multiple comparison test was used for statistical analysis. **P* < 0.05, ***P* < 0.01, ****P* < 0.001, *****P* < 0.0001, ns, not significant.


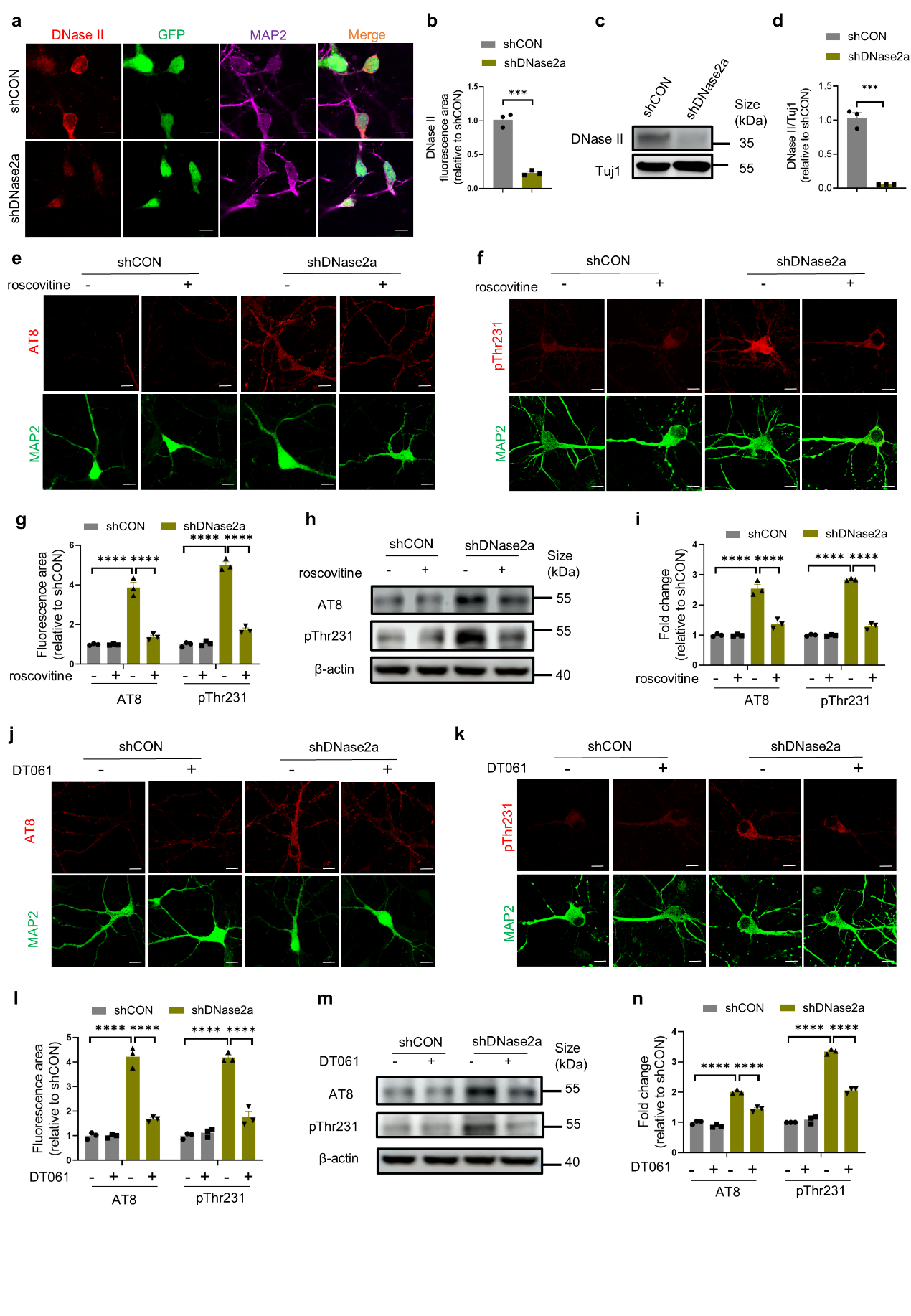


**Fig. S2** DNase Ⅱ deficiency induces tau phosphorylation by regulating kinases and phosphatases of tau in vitro. **a** Immunolabeling of DNase Ⅱ (red), GFP (green) and MAP2 (purple) in the primary hippocampal neurons infected with shDNase2a or shCON. Scale bars, 10 μm. **b** Quantification of DNase Ⅱ fluorescent area by Image J software in (**a**). **c** Western blotting of DNase Ⅱ levels in the primary hippocampal neurons infected with shDNase2a or shCON. **d** Quantitation of DNase Ⅱ levels in (**c**). **e** Immunolabeling of AT8 (red) and MAP2 (green) in the primary hippocampal neurons infected with shDNase2a or shCON with addition of CDK5 inhibitor roscovitine. Scale bars, 10 μm. **f** Immunolabeling of pThr231 (red) and MAP2 (green) in the primary hippocampal neurons infected with shDNase2a or shCON with addition of CDK5 inhibitor roscovitine. Scale bars, 10 μm. **g** Quantification of AT8 and pThr231 fluorescent areas in (**e**) and (**f**). **h** Western blotting of AT8 and pThr231 in the primary hippocampal neuron lysates infected with shDNase2a or shCON with addition of CDK5 inhibitor roscovitine. **i** Quantitation of the levels of AT8 and pThr231 in (**h**). **j** Immunolabeling of AT8 (red) and MAP2 (green) in the primary hippocampal neurons infected with shDNase2a or shCON with addition of PP2A agonist DT061. Scale bars, 10 μm. **k** Immunolabeling of pThr231 (red) and MAP2 (green) in the primary hippocampal neurons infected with shDNase2a or shCON with addition of PP2A agonist DT061. Scale bars, 10 μm. **l** Quantification of AT8 and pThr231 fluorescent areas in (**j**) and (**k**). **m** Western blotting of AT8 and pThr231 in the primary hippocampal neuron lysates infected with shDNase2a or shCON with addition of PP2A agonist DT061. **n** Quantitation the levels of AT8 and pThr231 in (**m**). In **a**, **c**, **e**, **f**, **h**, **j**, **k** and **m** data are representative of three independent experiments, and in **b** and **d**, data are pooled from three independent experiments. Data are mean ± SEM, and unpaired *t*-test with two-tailed *P* values was used for statistical analysis. In **g**, **i**, **l** and **n**, data are pooled from three independent experiments. Data are mean ± SEM, and a one-way ANOVA followed by Tukey’s multiple comparison test was used for statistical analysis. ****P* < 0.001, *****P* < 0.0001.


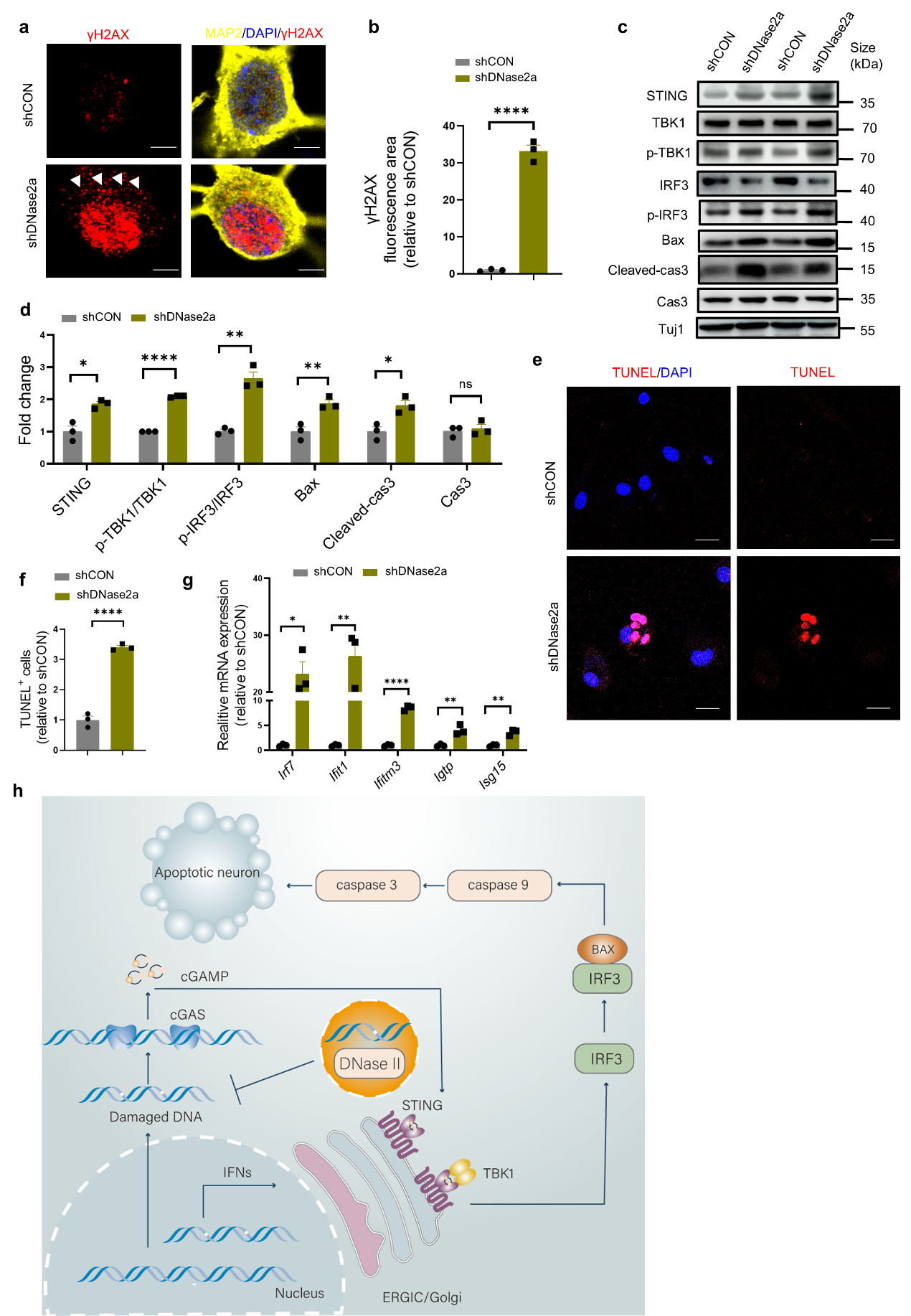


**Fig. S3** DNase Ⅱ downregulation induces neuronal apoptosis via cGAS-STING pathway. **a** Immunolabeling of γH2AX (red) and MAP2 (yellow) in the primary hippocampal neurons infected with shDNase2a or shCON, DAPI (blue). Scale bars, 3 μm. **b** Quantification of γH2AX fluorescent area by Image J software in (**a**). **c** Western blotting of STING, TBK1, p-TBK1, IRF3, p-IRF3, Bax, Cleaved-caspase 3 (Cleaved-cas3) and caspase 3 (cas3) in the primary hippocampal neurons infected with shDNase2a or shCON. **d** Quantitation of the bands in (**c**) by the Image J software. **e** Representative images of TUNEL (red) and DAPI (blue) in the primary hippocampal neurons infected with shDNase2a or shCON. Scale bars, 25 μm. **f** Quantification of TUNEL^+^ cells in (**e**). **g** qPCR analysis of mRNA expression of IFN-I related genes *Irf7*, *Ifit1*, *Ifitm3*, *Igtp* and *Isg15* in the primary hippocampal neurons infected with shDNase2a or shCON. **h** Schematic representation of signaling pathways involved in neuronal apoptosis via cGAS-STING pathway induced by DNase Ⅱ deficiency. In **a**, **c** and **e**, data are representative of three independent experiments, and in **b**, **d**, **f** and **g**, data are pooled from three independent experiments. Data are mean ± SEM, and unpaired *t*-test with two-tailed *P* values was used for statistical analysis. **P* < 0.05, ***P* < 0.01, *****P* < 0.0001.


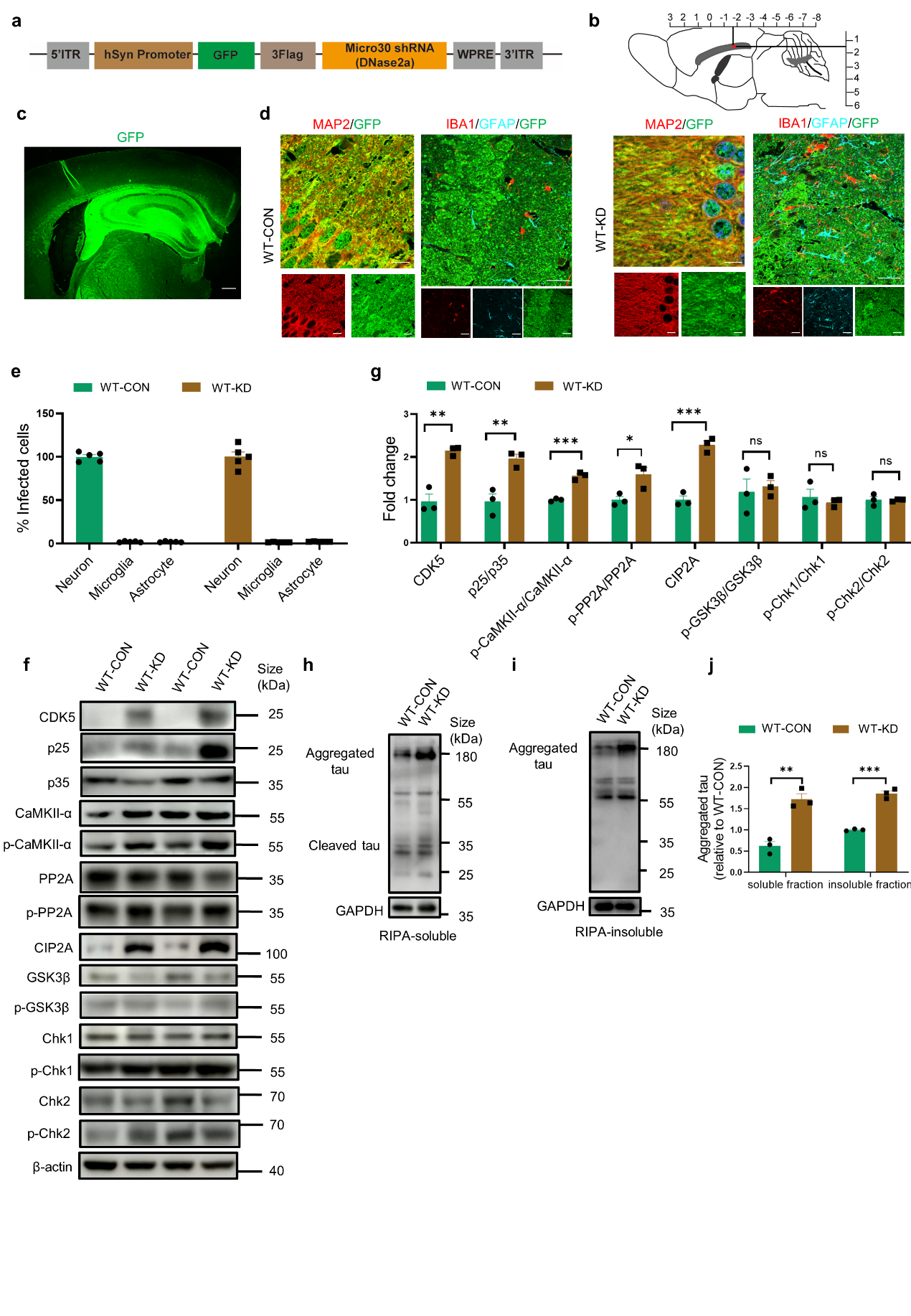


**Fig. S4** DNase Ⅱ deficiency increases tau phosphorylation and aggregation by regulating kinases and phosphatases of tau in vivo. **a** Schematic of DNase Ⅱ shRNA. **b** The stereotaxic injection site of mice for AAV delivery. **c** The distribution of GFP fluorescence in the brain of DNase Ⅱ-knockdown mouse model. Scale bars, 500 μm. **d** Immunolabeling of MAP2 (red), IBA1 (red) and GFAP (cyan) in GFP^+^ (green) cells in the brains of mice infected with AAV-shCON and AAV-shDNase2a. Scale bars, 8 μm or 25 μm. **e** Quantification of GFP fluorescence intensity in (**d**). *n* = 5 mice per group. **f** Western blotting of CDK5, p25, p35, CaMKII-α, p-CaMKII-α, PP2A, p-PP2A, CIP2A, GSK-3β, p-GSK-3β, Chk1, p-Chk1, Chk2 and p-Chk2 in the hippocampal homogenates of WT-CON mice or WT-KD mice. **g** Quantitation of the bands in (**f**) by the Image J software. **h, i** Western blotting of aggregated tau (detected using AT8) in RIPA-soluble and RIPA-insoluble hippocampal homogenates of WT-CON mice and WT-KD mice under non-reduced conditions. **j** Quantitation of the levels of aggregated tau in (**h**) and (**i**). In **f**, **h** and **i**, data are representative of three independent experiments, and in **g** and **j**, data are pooled from three independent experiments. Data are mean ± SEM, and unpaired *t*-test with two-tailed *P* values was used for statistical analysis. **P* < 0.05, ***P* < 0.01, ****P* < 0.001, ns, not significant.


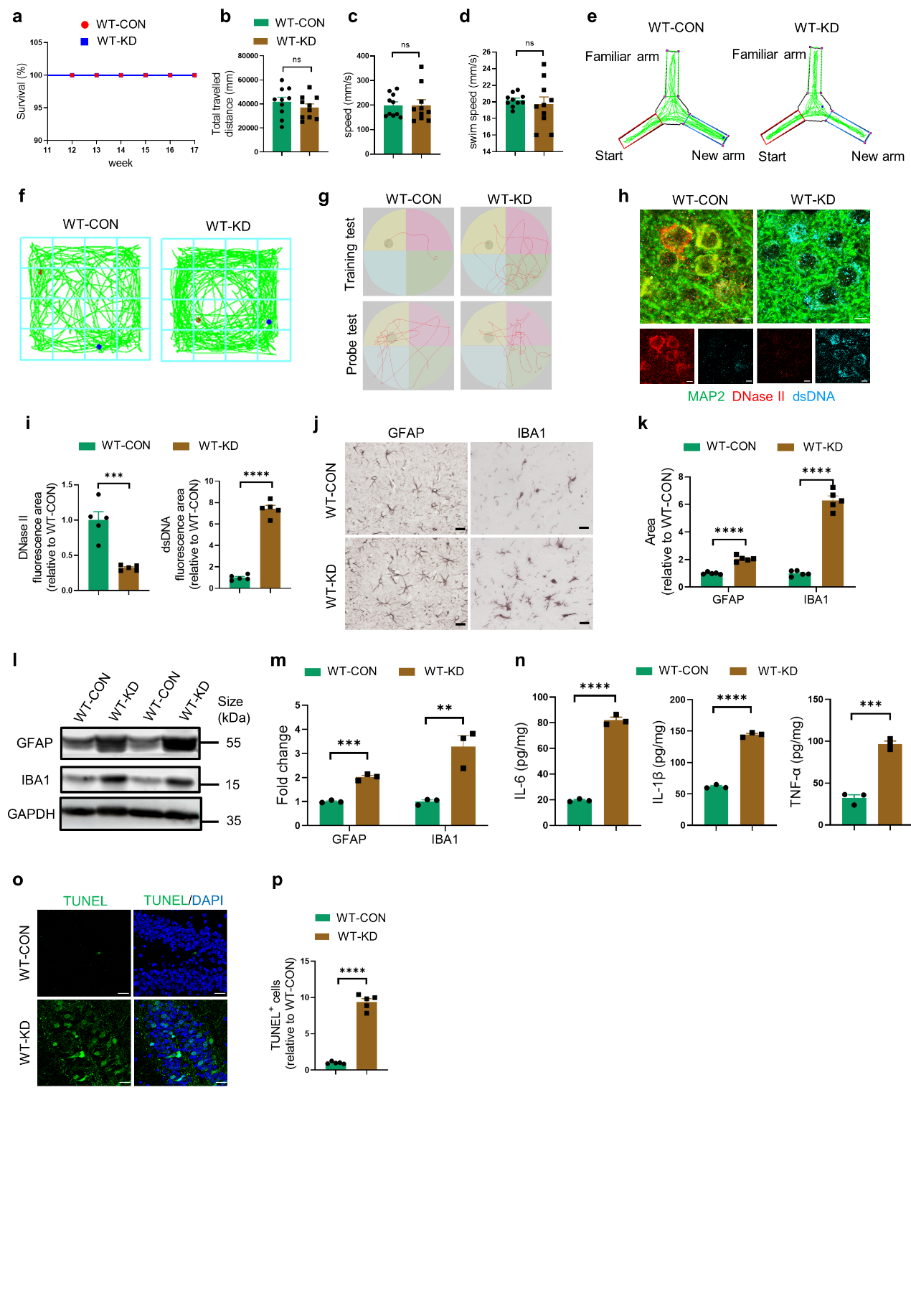


**Fig. S5** Neuronal DNase Ⅱ deficiency enhances neuroinflammation and apoptosis in 4-month-old WT mice. 3-month-old WT mice were treated with AAV-shCON and AAV-shDNase2a for 4 weeks, **a** the survival, **b** the total traveled distance in Y-maze and **c** speed in open field, **d** the swim speed in MWM test were measured. *n* = 10 mice per group. Data are mean ± SEM, and unpaired *t*-test with two-tailed *P* values was used for statistical analysis. **e** Representative 5-minute tracks of mice in forced Y-maze test. **f** Representative tracks of mice in open filed test. **g** Representative swimming paths in training test (up) and probe test (down) in MWM test. **h** Immunolabeling of DNase Ⅱ (red), dsDNA (cyan) and MAP2 (green) in the hippocampus of WT-CON mice and WT-KD mice. Scale bars, 3 μm. **i** Quantification of DNase Ⅱ and dsDNA fluorescent areas by Image J software. *n* = 5 mice per group. **j** Detection of astrocytes and microglia by immunolabeling GFAP and IBA1 in the hippocampus of WT-CON mice and WT-KD mice. Scale bars, 20 μm. **k** Quantification of GFAP, IBA1-labeled areas in (**j**). *n* = 5 mice per group. **l** Western blotting of GFAP and IBA1 in the hippocampus lysates of WT-CON mice and WT-KD mice. **m** Quantitation of the levels of GFAP and IBA1 in (**l**). **n** The levels of inflammatory cytokines IL-6, IL-1β and TNF-α in the hippocampus lysates of mice were measured by ELISA. **o** Representative images of TUNEL (green) and DAPI (blue) in hippocampus of mice. Scale bars, 50 μm. **p** Quantification of TUNEL^+^ cells in the hippocampus regions in (**o**). *n* = 5 mice per group. In **l**, data are representative of three independent experiments. In **m** and **n** data are pooled from three independent experiments. Data are mean ± SEM, and unpaired *t*-test with two-tailed *P* values was used for statistical analysis. ***P* < 0.01, ****P* < 0.001, *****P* < 0.0001, ns, not significant.


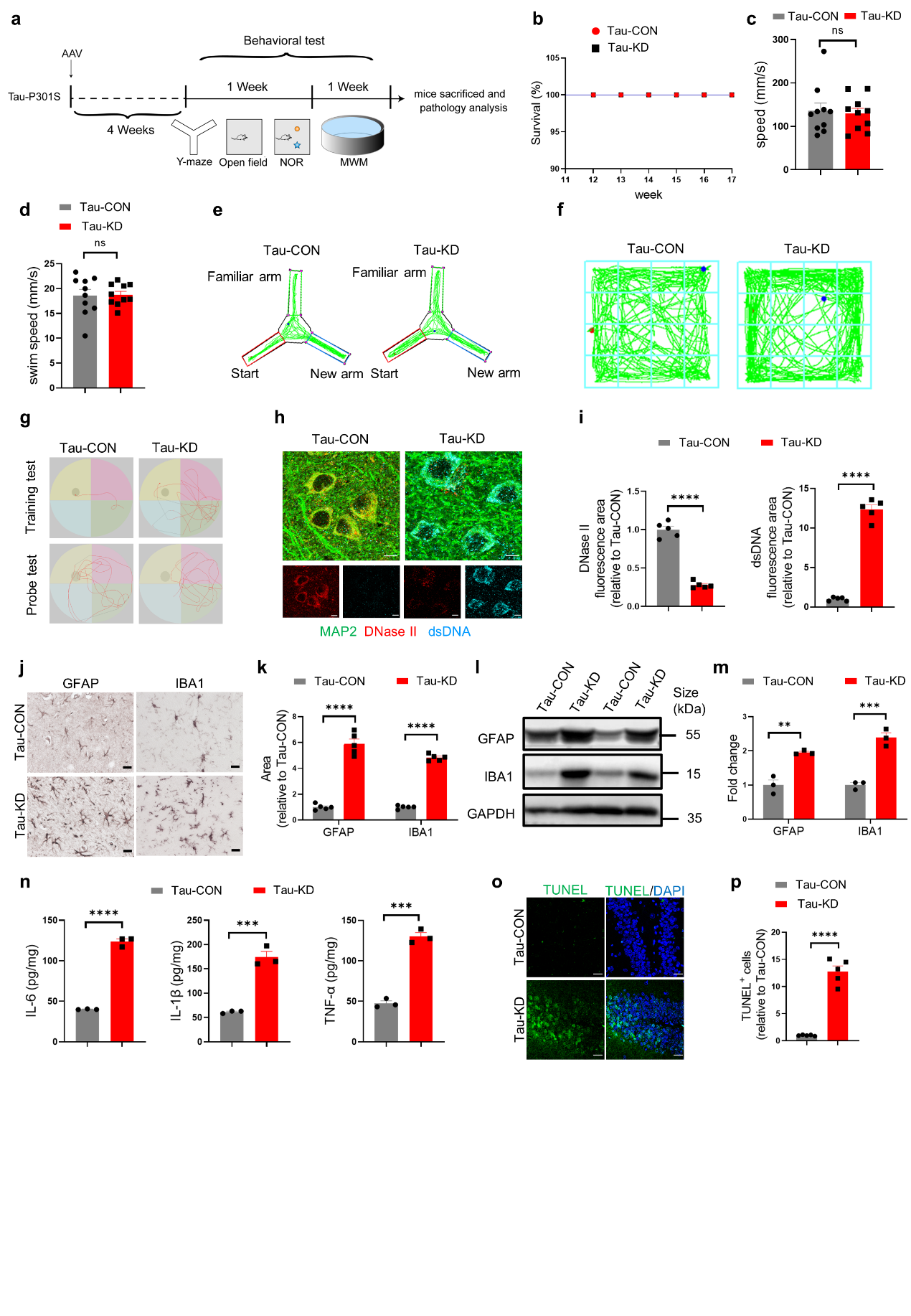


**Fig. S6** Neuronal DNase Ⅱ deficiency exacerbates gliosis and apoptosis in 4-month-old TauP301S mice. **a** Schematic representation of the pharmacological treatment and experimental measurement. The 3-month-old Tau-P301S mice were treated with AAV-shCON and AAV-shDNase2a for 4 weeks, **b** the survival, **c** speed in open field, **d** the swim speed in MWM test were measured. *n* = 10 mice per group. Data are mean ± SEM, and unpaired *t*-test with two-tailed *P* values was used for statistical analysis. **e** Representative 5-minute tracks of mice in forced Y-maze test. **f** Representative tracks of mice in open filed test. **g** Representative swimming paths in training test (up) and probe test (down) in MWM test. **h** Immunolabeling of DNase Ⅱ (red), dsDNA (cyan) and MAP2 (green) in the hippocampus of Tau-CON mice and Tau-KD mice. Scale bars, 3 μm. **i** Quantification of DNase Ⅱ and dsDNA fluorescent areas by Image J software in (**h**). *n* = 5 mice per group. **j** Detection of astrocytes and microglia by immunolabeling GFAP and IBA1 in the hippocampal region of Tau-CON mice and Tau-KD mice. Scale bars, 20 μm. **k** Quantification of GFAP, IBA1-labeled areas in (**j**). *n* = 5 mice per group. **l** Western blotting of GFAP and IBA1 in the hippocampus lysates of Tau-CON mice and Tau-KD mice. **m** Quantitation of the levels of GFAP and IBA1 in (**l**). **n** The levels of inflammatory cytokines IL-6, IL-1β and TNF-α in the hippocampus lysates of mice measured by ELISA. **o** Representative images of TUNEL (green) and DAPI (blue) in hippocampus of mice. Scale bars, 50 μm. **p** Quantification of TUNEL^+^ cells in the hippocampus regions in (**o**). *n* = 5 mice per group. In **l**, data are representative of three independent experiments. In **m** and **n**, data are pooled from three independent experiments. Data are mean ± SEM, and unpaired *t*-test with two-tailed *P* values was used for statistical analysis. ***P* < 0.01, ****P* < 0.001, *****P* < 0.0001, ns, not significant.


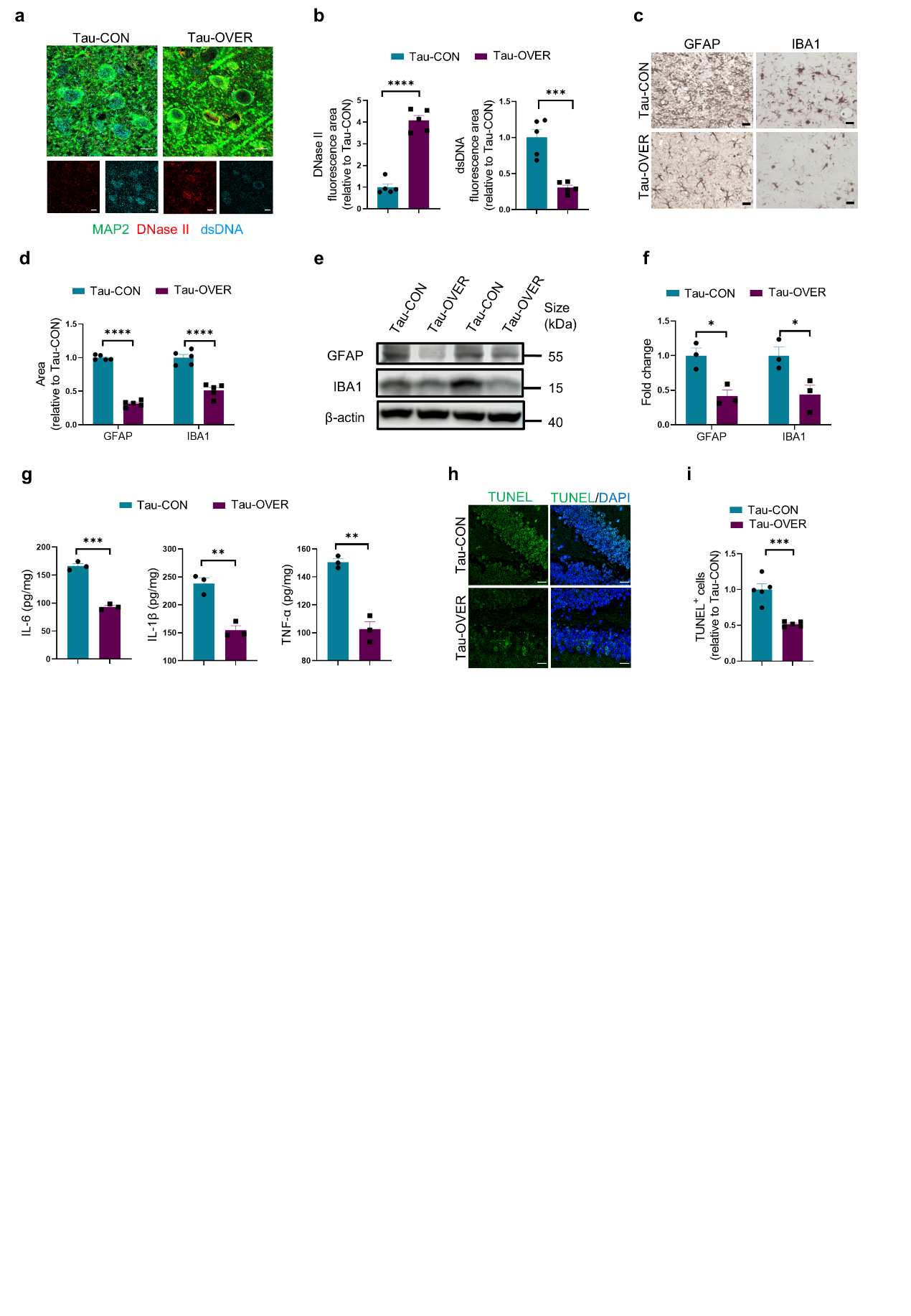


**Fig. S7** Neuronal DNase Ⅱ overexpression reduces neuroinflammation and apoptosis in Tau-P301S mice. **a** Immunolabeling of DNase Ⅱ (red), dsDNA (cyan) and MAP2 (green) in the hippocampus of Tau-CON mice and Tau-OVER mice. Scale bars, 3 μm. **b** Quantification of DNase Ⅱ and dsDNA fluorescent areas in (**a**) by Image J software. *n* = 5 mice per group. **c** Detection of astrocytes and microglia by immunolabeling GFAP and IBA1 in the hippocampal region of Tau-CON mice and Tau-OVER mice. Scale bars, 20 μm. **d** Quantification of GFAP, IBA1-labeled areas in (**c**). *n* = 5 mice per group. **e** Western blotting of GFAP and IBA1 in the hippocampal homogenates of Tau-CON mice and Tau-OVER mice. **f** Quantitation of the levels of GFAP and IBA1 in (**e**). **g** The levels of inflammatory cytokines IL-6, IL-1β and TNF-α in the hippocampus lysates of mice measured by ELISA. **h** Representative images of TUNEL (green) and DAPI (blue) in hippocampus of Tau-CON mice and Tau-OVER mice. Scale bars, 50 μm. **i** Quantification of TUNEL^+^ cells in the hippocampus regions in (**h**). *n* = 5 mice per group. In **e**, data are representative of three independent experiments. In **f** and **g** data are pooled from three independent experiments. Data are mean ± SEM, and unpaired *t*-test with two-tailed *P* values was used for statistical analysis. **P* < 0.05, ***P* < 0.01, ****P* < 0.001, *****P* < 0.0001.

**Table S1.** Demographic details of the plasma used in this study.

| Case | Age | Gender | Clinical diagnosis | Case | Age | Gender | Clinical diagnosis |
| --- | --- | --- | --- | --- | --- | --- | --- |
| 1 | 66 | M | MCI | 22 | 81 | F | AD |
| 2 | 57 | F | MCI | 23 | 80 | M | AD |
| 3 | 76 | M | MCI | 24 | 60 | M | AD |
| 4 | 59 | M | MCI | 25 | 84 | F | AD |
| 5 | 71 | F | MCI | 26 | 75 | F | AD |
| 6 | 84 | F | MCI | 27 | 60 | F | AD |
| 7 | 82 | F | MCI | 28 | 85 | F | AD |
| 8 | 73 | M | MCI | 29 | 64 | M | AD |
| 9 | 82 | F | MCI | 30 | 70 | M | Normal |
| 10 | 76 | F | MCI | 31 | 74 | F | Normal |
| 11 | 64 | M | MCI | 32 | 65 | M | Normal |
| 12 | 75 | F | MCI | 33 | 76 | M | Normal |
| 13 | 73 | M | MCI | 34 | 65 | F | Normal |
| 14 | 71 | F | MCI | 35 | 71 | F | Normal |
| 15 | 70 | F | AD | 36 | 71 | M | Normal |
| 16 | 57 | F | AD | 37 | 70 | M | Normal |
| 17 | 74 | M | AD | 38 | 65 | F | Normal |
| 18 | 76 | M | AD | 39 | 76 | M | Normal |
| 19 | 72 | M | AD | 40 | 69 | F | Normal |
| 20 | 69 | M | AD | 41 | 78 | M | Normal |
| 21 | 57 | F | AD | 42 | 72 | M | Normal |

MCI, mild cognitive impairment; AD, Alzheimer’s disease; M, male; F, female.

**Table S2.** Primer sequences.

| **Gene** | **Forward 5’-3’** | **Reverse 5’-3’** |
| --- | --- | --- |
| *mβ-actin* | TGTGATGGTGGGAATGGGTCAG | TTTGATGTCACGCACGATTTCC |
| *mIrf7* | AAATAGGGAAGAAGTGAGCCTC | CCCTTGTACATGATGGTCACAT |
| *mIfit1* | AGAGTCAAGGCAGGTTTCTG | TGTGAAGTGACATCTCAGCTG |
| *mIfitm3* | CTTCATAGCCTATGCCTACTCC | TGTGAAGGTTTTGAGCGTTAAG |
| *mIgtp* | CTCATCAGCCCGTGGTCTAAA | CACCGCCTTACCAATATCTTCAA |
| *mIsg15* | CTAGAGCTAGAGCCTGCAG | AGTTAGTCACGGACACCAG |
